# Steric Control in Low-Valent Mn Diamide Complexes: Contrasting Magnesium and Manganese in N_2_ and Benzene Activation

**DOI:** 10.1021/jacs.5c08422

**Published:** 2025-07-10

**Authors:** Siad Wolff, Matthew J. Evans, Thayalan Rajeshkumar, Dat T. Nguyen, Konstantin B. Krause, Amanda Opis-Basilio, Christian Herwig, Laurent Maron, Cameron Jones, Christian Limberg

**Affiliations:** † Institut für Chemie, 9373 Humboldt-Universität zu Berlin, Brook-Taylor-Straße 2, 12489 Berlin, Germany; ‡ School of Chemistry, Monash University, PO Box 23, Melbourne, Victoria 3800, Australia; § 27091 Université de Toulouse et CNRS, INSA, UPS, UMR5215, LPCNO, 135 Avenue de Rangueil, 31077 Toulouse, France

## Abstract

Reduction of Mn^II^ precursors with bulky diamide ligands provided access to a complex with the longest known Mn–Mn bond and to a rare example of N_2_ activation at high-spin Mn^I^ centers. While some instructive parallels can thus be drawn to observations made for Mg analogues, the accessibility of filled d orbitals in the respective Mn^I^ intermediates leads to a distinct behavior toward benzene that undergoes an oxidative addition.

## Graphical abstract

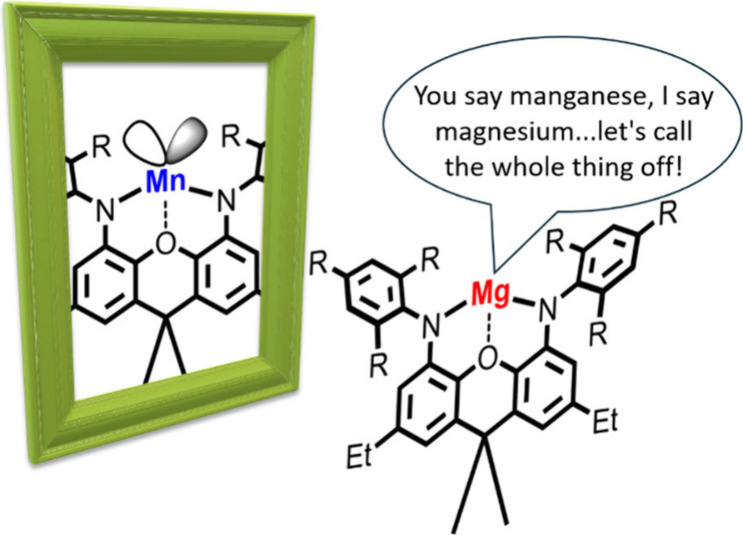

Despite the rich chemistry of transition metal complexes used for N_2_ fixation,^1–3^ only a few examples are known that utilize manganese.^4^ In fact, most manganese complexes that are capable of binding N_2_ feature low-spin manganese sites equipped with carbonyl or cyclopentadienyl ligands. In case of high-spin systems, only two examples have been reported so far.^5,6^

Successful N_2_ binding requires reduction of a Mn^II^ precursor, and it has been frequently observed that the resulting high-spin Mn^I^ species dimerize under formation of Mn–Mn bonds.^7^ The first example was the reduction of a β-diketiminate Mn^II^ precursor leading to the dinuclear complex **I** with a Mn–Mn bond (Figure 1).^8^ This observation was in contrast to the behavior of other low-valent β-diketiminate first row transition metal species (Cr, Fe–Ni), which bind N_2_ upon reduction.^9–12^ Calculations revealed that both Mn^I^ centers within **I** exhibit an electron configuration of [3d^5^4s^1^4p^0^], whereas the Mn–Mn bond is mainly formed by the overlap of the 4s orbitals. Since then, further representatives with a Mn_2_^2+^ core have been reported, indicating that formation of a metal–metal bond is a universal phenomenon for low-coordinated high-spin Mn^I^ centers.^13–16^ Although these systems are still able to activate certain small molecules,^8,17^ formation of the Mn–Mn bond blocks reactivity toward N_2_ by pairing the high energetic 4s electrons.

**Figure 1:**
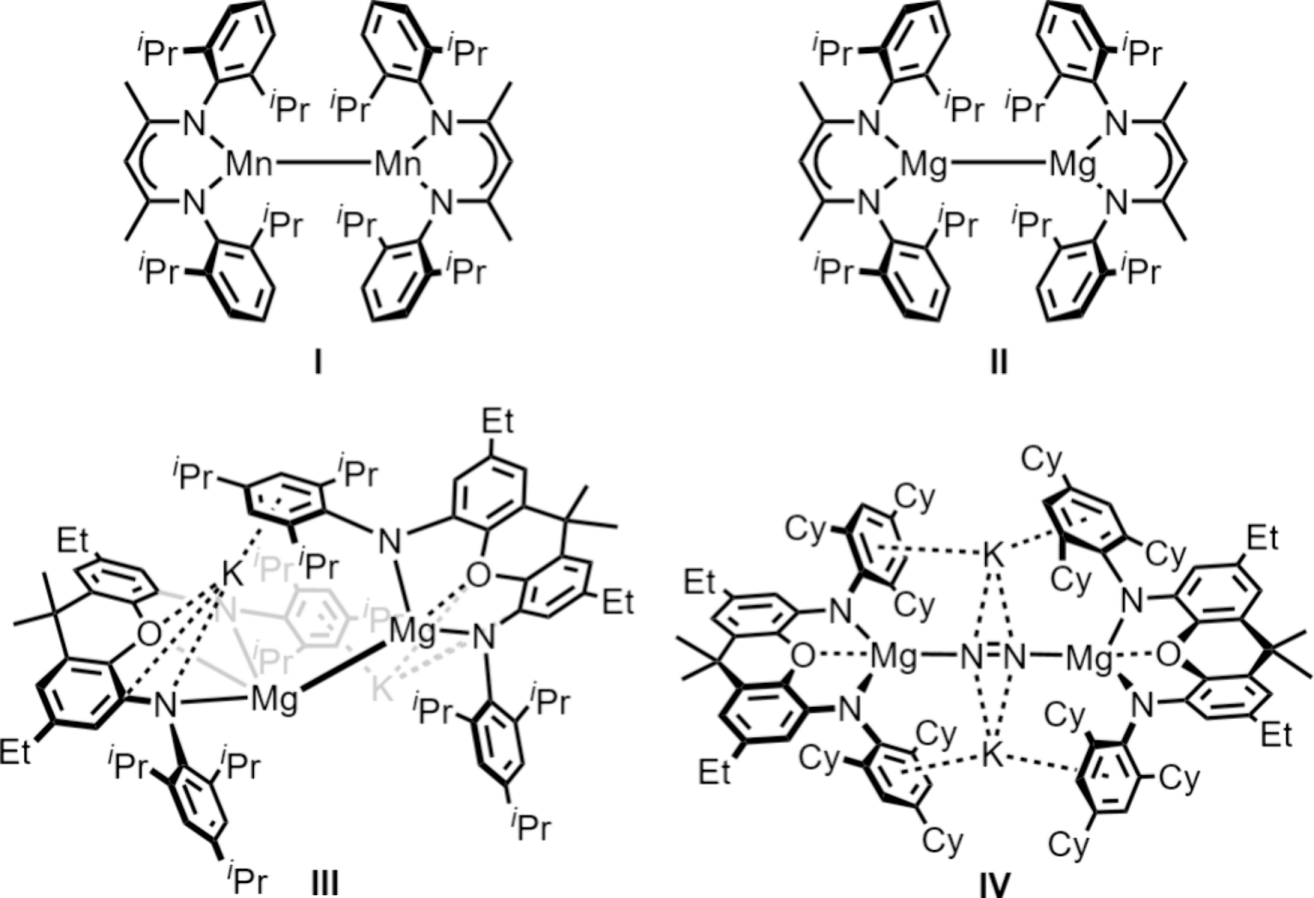
Isostructural β-diketiminate dinuclear metal­(I) compounds **I** and **II** and the recently reported Mg^I^ or masked Mg^I^ complexes **III** and **IV** stabilized by bulky xanthene-based diamide ligands.

Similarly, also for low-valent Mg^I^ complexes, formation of a Mg–Mg bond is observed, driven by the overlap of the singly occupied 3s orbitals.^18^ One of the very first examples of Mg^I^ dimers was accessed using β-diketiminate coligands, namely, the complex **II** reported in 2007 (Figure 1),^19^ which is isostructural to **I**. While Mg^I^ dimers are nowadays widely employed as versatile reductants in organometallic chemistry,^20,21^ much effort has been spent to prevent Mg–Mg bond formation by using superbulky β-diketiminate ligands in order to allow generation of even more reactive Mg^I^ radicals.^22–28^

Following the recent pioneering work on calcium mediated N_2_ activation,^29,30^ some of us reported that potassium reduction of [Mg­(^Trip^NON)] (^Trip^NON = 4,5-bis­(2,4,6-triisopropylanilido)-2,7-diethyl-9,9-dimethyl-xanthene) gives rise to a dimagnesium­(I)/dipotassium­(I) complex **III** (Figure 1), featuring a very long Mg–Mg bond, a result of the steric repulsion of the bulky Trip arene substituents.^31^ When the magnesium diamide precursor [Mg­(^TCHP^NON)] (^TCHP^NON = 4,5-bis­(2,4,6-tricyclohexylanilido)-2,7-diethyl-9,9-dimethyl-xanthene) was reduced, steric frustration induced by the even bulkier TCHP arene substituents prevented the homocoupling of transient Mg^I^ radicals and allowed reduction of N_2_, which got bound as a bridging diazene ligand (**IV**, Figure 1).^32^ Given the similar challenges in the chemistry of low-valent Mg and Mn, we were interested in applying the palette of superbulky diamide ligands toward Mn to achieve N_2_ activation.^33^

Synthesis of the Mn^II^ diamido precursors was achieved via salt-metathesis of the respective lithium amide salts with MnCl_2_ in THF, yielding [(^Trip^NON)­Mn­(THF)_2_], **1**, and [(^TCHP^NON)­Mn­(THF)], **2** (Scheme 1). The molecular structure of **1** as determined by single crystal XRD revealed the coordination of two THF molecules, while for **2**, coordination of just one THF ligand to give an overall distorted square-planar ligand arrangement (τ^4^ = 0.43) was observed (ESI Figures S14 and S15). The steric maps, constructed based on the X-ray diffraction data by using the web application SambVca 2,^34^ visualize that for **2** the THCP substituents enclose the metal such that no coordination in axial position is possible (Figure 2). Both **1** and **2** are highly air sensitive high-spin Mn^II^ systems and were spectroscopically fully characterized (see ESI for details).

**Figure 2:**
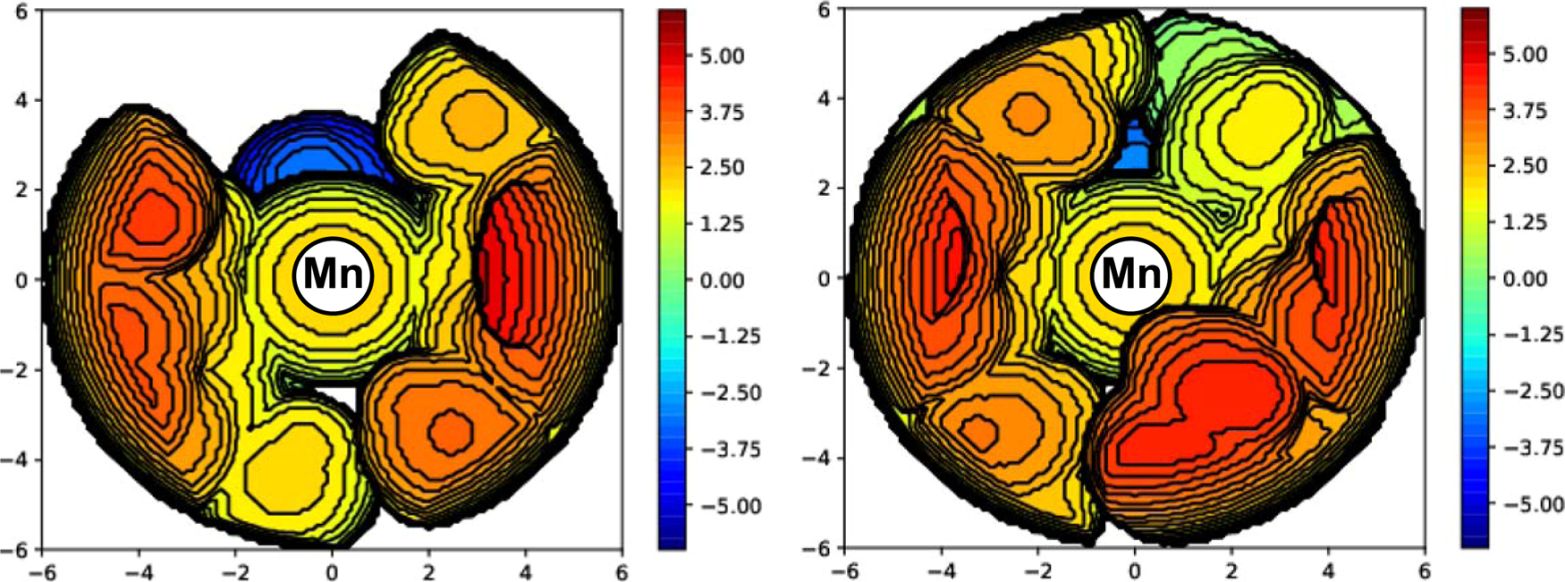
Topographic steric maps of **1** (left, buried volume *V*_B_ = 57.3%) and **2** (right, buried volume *V*_B_ = 68.8%) with a sphere diameter of 6 Å shown along the Mn–O_Xanthen_ bond, omitting the THF ligands (H atoms were included during modeling).

**Scheme 1:**
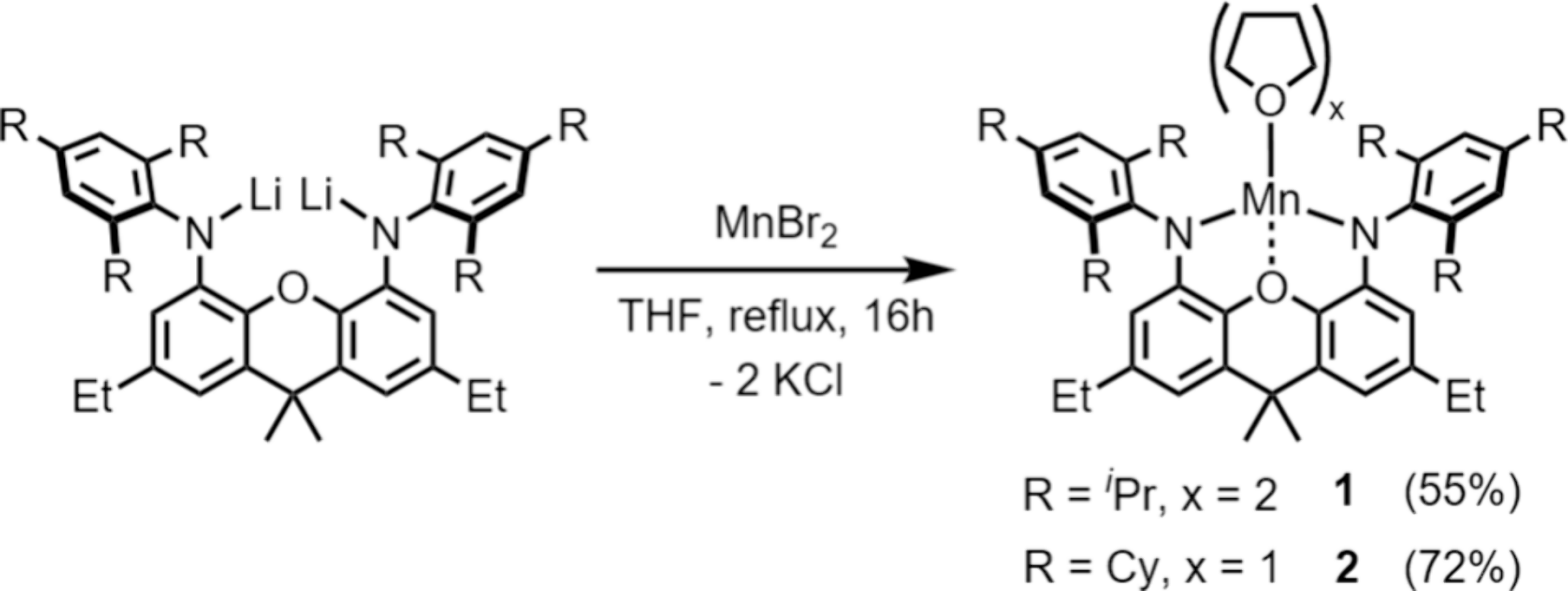
Synthesis of Compounds **1** and **2**

Potassium reduction of **1** with an excess of 5% w/w K/KI^35^ (under an N_2_ atmosphere) gave the dimanganese­(I) complex [(K­(^Trip^NON)­Mn)_2_], **3**, as a red solid in 28% yield (Scheme 2). Single crystal X-ray analysis confirmed that **3** is isostructural to the previously reported Mg analogue **III** (Figure 3), with similar bonding parameters of the metal centers toward the NON-ligand. Thereby **3** and **III** feature the same asymmetric coordination of the K^+^ counterions. The Mn centers within **3** are directly connected by a Mn–Mn bond at a distance of 2.9497(7) Å. This bond is slightly shorter than the Mg–Mg bond within **III** (3.137(2) Å). Magnetic measurements revealed, at room temperature, an effective magnetic moment of μ_eff_ = 8.00 μ_B_ (close to the value expected for two uncoupled S = 2.5 Mn centers of 8.36 μ_B_), which decreases upon cooling, thus indicating antiferromagnetic coupling. DFT calculations including dispersion corrections (see ESI for details) on complex **3** rationalized the bonding situation as well as the (Mn^I^)_2_ electron configuration as follows: **3** has an open-shell singlet ground state (5 unpaired electrons per Mn, antiferromagnetically coupled) and the Mn–Mn single bond is covalent (50–50 contribution of the two Mn atoms), formed with sd hybrid atomic orbitals (60% to 80% s character). This is in line with the result of the SQUID measurement, which shows a decrease of the magnetic moment with *T* → 0 K, and the simulation as well as DFT analysis both gave small negative *J* values, characteristic for weak antiferromagnetic coupling (see ESI).

**Scheme 2:**
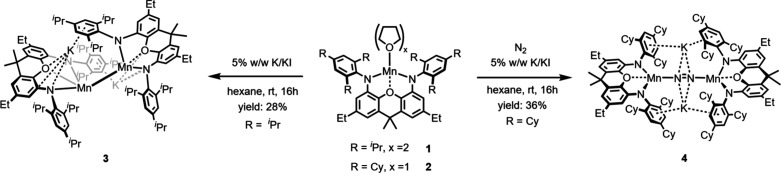
Synthesis of Compounds **3** and **4**

**Figure 3:**
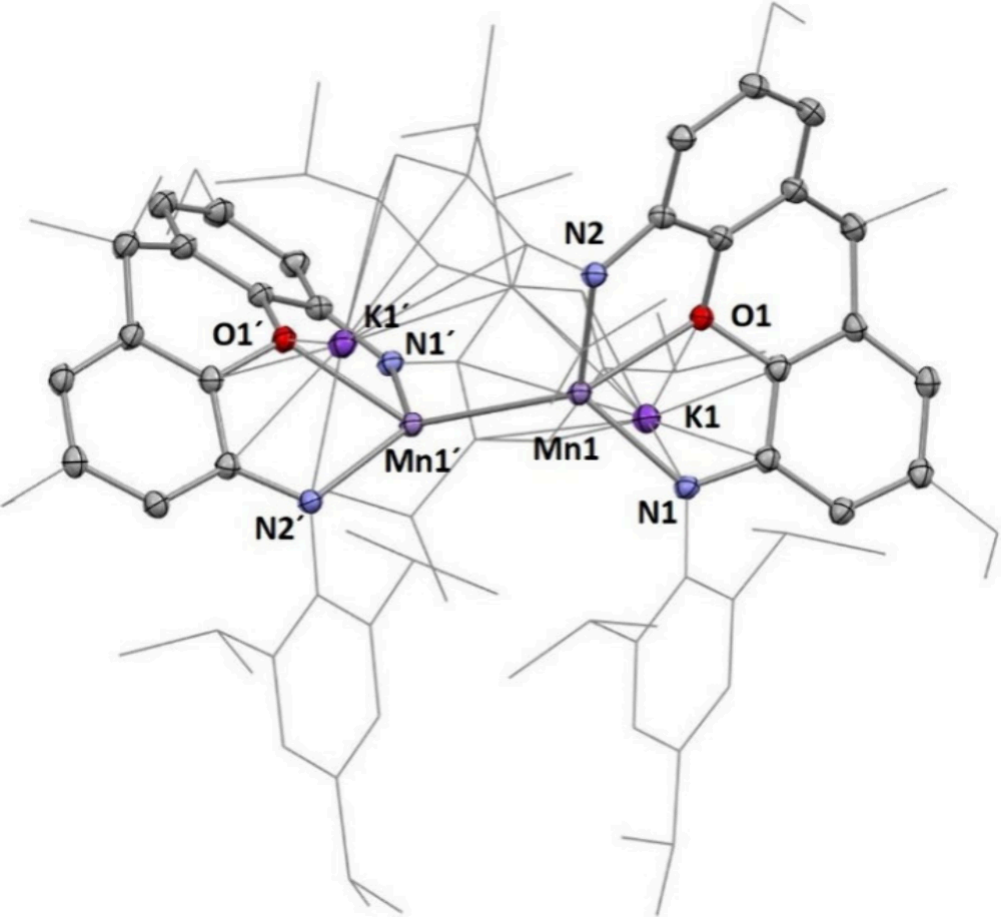
Molecular structure of **3** as determined by X-ray diffraction analysis. Thermal ellipsoids are shown at the 50% probability level. H atoms have been omitted for the sake of clarity. Selected bond lengths (Å) and angles (°): Mn1–O1, 2.318(2); Mn1–N1, 2.188(2); Mn1–N2, 2.146(2); Mn1–Mn1′, 2.9497(7); Mn1′–Mn1–O1, 125.84(5); N1–Mn1–N2, 123.30(8). Geometry index τ^4^_Mn1_ = 0.78, τ^4^_Mn1_´ = 0.78.

Comparison of **3** with related high-spin Mn^I^ dimers shows that to the best of our knowledge **3** features the longest Mn–Mn bond reported so far,^7,8,13–16^ but similarly as in the case of the corresponding Mg^I^ compound, the Trip arene substituents are not sufficiently bulky to prevent metal–metal bond formation. However, when using the sterically even more demanding ^TCHP^NON ligand, potassium reduction of **2** under an N_2_ atmosphere afforded the dinuclear dinitrogen complex [(K­(^TCHP^NON)­Mn)_2_(μ-η^1^:η^1^-N_2_)], **4**, as an orange solid in 36% yield. The molecular structure determined via single crystal X-ray diffraction confirmed that **4** is again isostructural to the previously described dimagnesium­(II) diazene complex **IV** (Figure 4). Two (^TCHP^NON)­Mn^II^ moieties are spanning an N_2_^2–^ unit in an end-on fashion, while there are side-on contacts with two K^+^ ions. Both Mn centers exhibit distorted square-planar coordination spheres (τ^4^ = 0.47), as also observed for the precursor compound **2**. The N–N distance in **4** (1.226(5) Å) is slightly shorter than that observed within **IV** (1.255(3) Å) but is still considerably elongated compared with the bond in N_2_ (1.098 Å) and also compared to the two known systems reported by Arnold et al.^5^ (d­(NN) = 1.208(6) Å) and Theopold et al.^6^ (d­(NN) = 1.196(5) Å). Moreover, unlike in the two latter representatives, the dinitrogen activating moiety in **4** is anionic.

**Figure 4:**
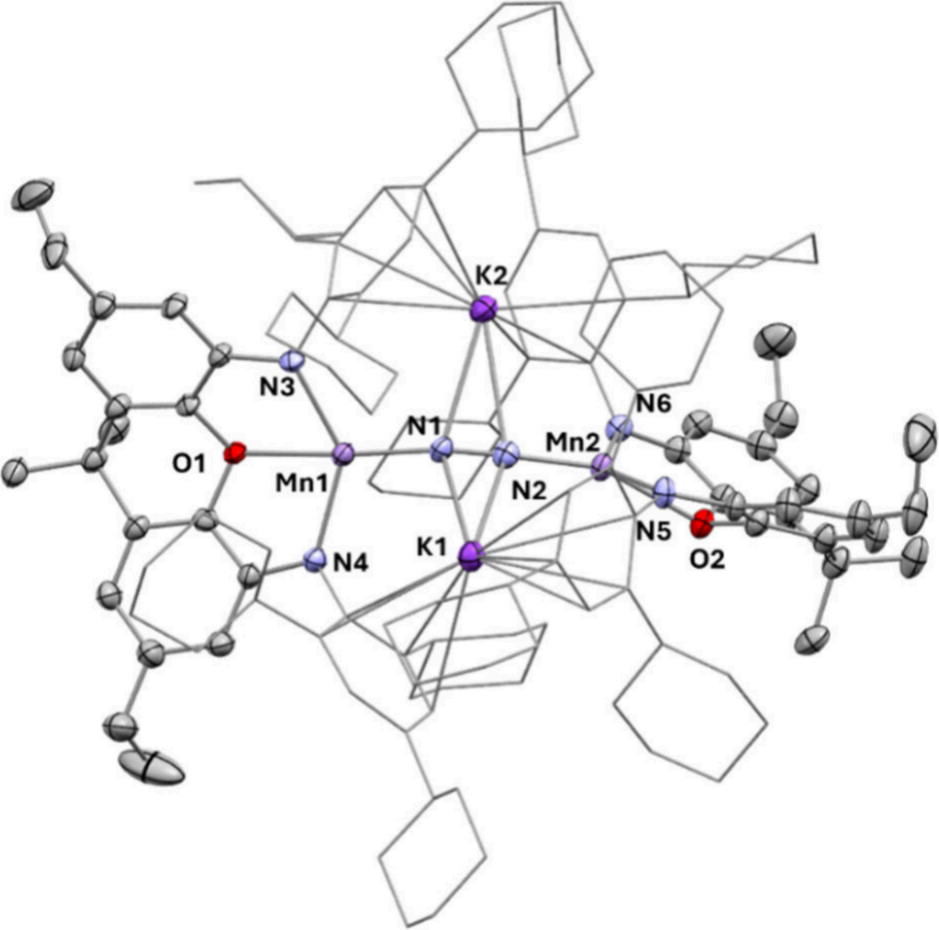
Molecular structure of **4** as determined by X-ray diffraction analysis. Thermal ellipsoids are shown at the 50% probability level. H atoms and cocrystallized solvent molecules are omitted for clarity. Selected bond lengths (Å) and angles (°): N1–N2, 1.226(5); Mn1–O1, 2.331(3); Mn2–O2, 2.303(3), Mn1–N1, 1.880(4); Mn2–N2, 1.872(4); Mn1–N3, 2.090(4); Mn1–N4, 2.068(5); Mn2–N5, 2.107(3); Mn2–N6, 2.096(4); N3–Mn1–N4, 136.1(2); N5–Mn2–N6, 136.6(1). Geometry index τ^4^_Mn1_ = τ^4^_Mn2_ = 0.47, τ^4^_Mn1_´ = τ^4^_Mn2_´ = 0.40.

The Raman spectrum of **4** displays an N–N stretching band at ν = 1615 cm^–1^, that is, at a significantly higher wavenumber than the band of **IV** (ν = 1530 cm^–1^), but still indicating “strong” ^2,36^ activation. Hence, the N_2_ ligand in **IV** is activated more strongly than the one in its Mn derivative, which likely originates in the complete electron transfer from the reduced Mg center in combination with the lack of π back bonding. This results in a significant accumulation of negative charge on the diazenide ligand in **IV**.

Hence, both the Raman spectroscopic data as well as the N–N distance indicate that **4** contains a N_2_^2–^ ligand coordinated to two Mn^II^ centers, which is also in line with the results of magnetic measurements: Evans NMR and SQUID measurements revealed an effective magnetic moment of μ_eff_ = 8.48 μ_B_ at room temperature for **4**, both in the solid-state and in solution. The SQUID measurement moreover showed that the magnetic moment is temperature independent, indicating a strong coupling of the manganese centers. This is mediated by the diazenide ligand, which has two unpaired electrons with parallel spins, to which the Mn spins are coupled antiferromagnetically so that altogether their spins are coupled ferromagnetically. Hence, 10 parallel unpaired spins result for the two Mn^II^ ions, two of which are canceled by the diazenide, matching the result of the SQUID measurement (μ_s.o._ for S = 4 is 8.94 μ_B_). As for complex **3**, DFT calculations using the same level of theory were carried out on complex **4**. Indeed, the nonet spin state (S = 4) was identified as the ground state (see ESI Table S7). The optimized geometry agrees excellently with the experimental data, as exemplified by the N–N distance, which is perfectly reproduced (1.22 Å). The unpaired spin values (see ESI Table S8) indicate the presence of 5 alpha spins on each Mn while two beta spins are located at the N_2_^2–^ unit.

We have previously found that complex **IV** can be considered as a “masked” dimagnesium­(I) diradical species^32,37^ that is able to react with benzene in “Birch-type” reductions affording the complex **V** with concomitant release of the N_2_ ligand (see Scheme 3).^38^ The high reactivity of **IV** toward benzene and other substrates, that are usually considered as inert, derives from labile binding of the N_2_^2–^ ligand: Upon contact with molecules, which have better ligand donor/electron accepting properties, in a formal view, the original diazene unit transfers its two electrons to the metal centers with elimination of N_2_. The substrate that has triggered this process then gets activated at the reduced metal centers. However, for **4**, significantly shorter metal–N_2_ distances are observed as compared to **IV** (**4**: 1.880(4)/1.872(4) Å vs **IV**: 1.987(2)/1.993(2) Å), indicative of a stronger binding of the N_2_^2–^ ligand resulting from higher degrees of π-back bonding. This observation also rationalizes the fact that **4** is stable toward aromatic solvents and ethers, whereas **IV** readily decomposes in such environments. Hypothesizing that arene reduction may be achieved by the in situ generated Mn^I^ species that activates N_2_ to give **4**, reduction of **2** was repeated in benzene solution in the absence of N_2_. Indeed, a reaction with benzene occurred, yielding, however, not a “Birch-type” product but a Mn^III^ phenyl complex [(^TCHP^NON)­Mn­(Ph)], **5**, as confirmed by single crystal X-ray diffraction analysis (ESI Figure S16). The Mn–NON–ligand bonds within **5** are slightly shorter compared to the ones observed for **2**, supporting the formulation of a Mn^III^ complex. Magnetic measurements of **5** revealed an effective magnetic moment of μ_eff_ = 4.31 μ_B_, characteristic for a high-spin Mn^III^ center.

**Scheme 3:**
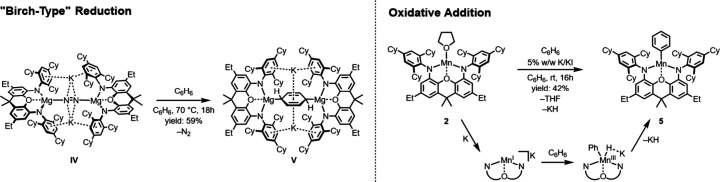
Arene Reduction Mediated by Low-Valent Mg and Mn ^TCHP^NON Complexes

The formation of both, a Ph^–^ ligand as well as the Mn^III^ center, may be rationalized by an initial oxidative addition. We suggest that the nonstabilized Mn^I^ complex [K­(^TCHP^NON)­Mn] generated upon potassium reduction of **2** is sufficiently reduced to insert into an aromatic C–H bond. The potential intermediate [K­(^TCHP^NON)­MnH­(Ph)] thus resulting then eliminates KH to give complex **5**, most likely caused by the steric demand around the Mn^III^ site and the very high lattice energy of generated KH. Hence, the ability to access higher oxidation states and the availability of π-symmetric filled d orbitals allows [K­(^TCHP^NON)­Mn] to perform an oxidative addition of benzene while the Mg derivative [K­(^TCHP^NON)­Mg] is limited to 1e^–^ transfer reactions per Mg center yielding the “Birch-type” product **V**. The formation of complex **5** from complex **2** was thus investigated computationally at the same level of theory as before (Figure 5). In the presence of potassium as a reducing agent and benzene, the formation of a stable intermediate **Int1** is proposed. In this intermediate, the benzene ring is bound through its π system between the Mn center and the potassium cation. Upon activation, one aromatic C–H bond is readily cleaved via the transition state **TS1** with an activation barrier of 15.6 kcal/mol in enthalpy (18.5 kcal/mol in Gibbs Free energy). At **TS1**, the C–H bond is already broken (1.62 Å) and the Mn–C bond is being formed (2.06 Å). Notably, the Mn–H distance is short (1.62 Å), consistent with an oxidative addition reaction. Following the intrinsic reaction coordinate, a quite unstable intermediate **Int2** is yielded (+1.8 kcal/mol with respect to the separated reactant in enthalpy, 7.1 kcal/mol in Gibbs free energy). In the last step, the elimination of KH from the coordination sphere is occurring. It is interesting to note that the formation of a KH molecule in solution is very unlikely since this step is endothermic by more than 40 kcal/mol. On the other hand, the formation of KH in the solid-state, with a very high lattice energy of 172.6 kcal/mol, drives the reaction toward complex **5**. Hence, the reaction of [K­(^TCHP^NON)­Mn] with benzene to give **5** likely proceeds via an oxidative addition, which so far has been rarely observed in manganese chemistry.^39,40^

**Figure 5:**
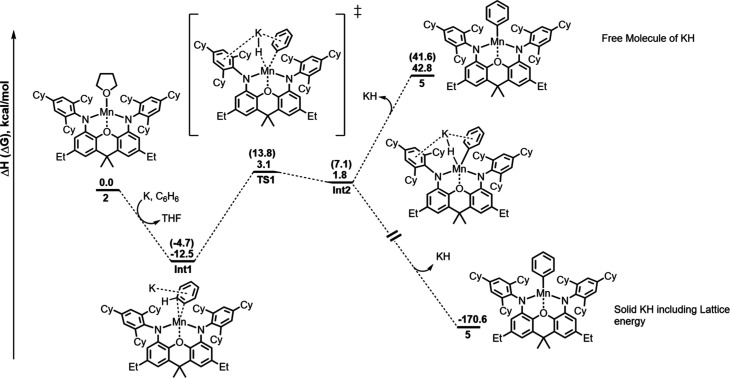
Computed enthalpy (Gibbs Free energy between bracket) reaction profile for the formation of complex **5** from the reduction of complex **2** in the presence of benzene at room temperature. The energies are given in kcal/mol.

In summary, we have shown that super bulky xanthene-based diamide ligands allow steric control over metal–metal bond formation vs N_2_ activation, not only for Mg as the central metal ion but also for Mn, which highlights the similarities between both metals. However, the fact that Mn is a transition metal also leads to distinct behavior in reactivity between both systems. While **IV** performs “Birch-type” reductions of arenes, via transient Mg^I^ radicals [K­(^TCHP^NON)­Mg], the corresponding analogue **4** is unreactive toward aromatic compounds, as the diazene unit is bound more strongly. However, in situ-generated [K­(^TCHP^NON)­Mn] does react with benzene, in contrast to [K­(^TCHP^NON)­Mg], not in a “Birch-type” reduction but via oxidative addition of C–H bonds due to the accessibility of the + III oxidation state. The ability of the Mn center in **4** to participate in π-back bonding leads to a higher stability of the complex (compared to **IV**), which might enable N_2_ functionalization in future studies.

## Supplementary Material





## Notes

Full synthetic, crystallographic, and computational details as well as spectroscopic data for new compounds (PDF)
Coordinates of the calculated structures (XYZ)
