# Supplementary material for: Steric Control in Low-Valent Mn Diamide Complexes: Contrasting Magnesium and Manganese in N2 and Benzene Activation
Source: J Am Chem Soc. 2025 Jul 10;147(29):25174–9. doi: 10.1021/jacs.5c08422 (PMC12291459; doi:10.1021/jacs.5c08422)
Supplement: Supplementary file 1 [file ja5c08422_si_001.pdf]

Supporting Information

Steric Control in Low-Valent Mn Diamide Complexes: Contrasting Magnesium and Manganese in N_2 and Benzene Activation

Siad Wolff,[a] Matthew J. Evans,[b] Thayalan Rajeshkumar,[c] Dat T. Nguyen,[b] Konstantin B. Krause,[a] Amanda Opis-Basilio,[a] Christian Herwig,[a] Laurent Maron,[c]* Cameron Jones,[b]* Christian Limberg[a]*

[a]Institut für Chemie, Humboldt-Universität zu Berlin, Brook-Taylor-Straße 2, 12489 Berlin, Germany.

[b]School of Chemistry, Monash University, Melbourne, PO Box 23, Victoria, 3800, Australia.

[c]Laboratoire de Physique et Chimie des Nanoobjets, Université de Toulouse, INSA-CNRS, 135, avenue de Rangueil, 31077 Toulouse, France.

E-Mail: christian.limberg@chemie.hu-berlin.de
cameron.jones@monash.edu
laurent.maron@irsamc.ups-tlse.fr

Table of content

1. General Considerations. 2

2. Synthetic procedures 3

2.1 Synthesis of [(^{Trip}NON)Mn(THF)$_2$], 1 3

2.2 Synthesis of [(^{TCHP}NON)MnTHF], 2 4

2.3 Synthesis of [(K(^{Trip}NON)Mn)$_2$], 3 5

2.4 Synthesis of [(K(^{TCHP}NON)Mn)$_2$(μ-η^1:η^1-N$_2$)], 4 6

2.5 Synthesis of [(^{TCHP}NON)Mn(C$_6$H$_5$)], 5 8

3. SQUID Measurements 9

4. EPR Measurements 12

5. Crystallographic Data 14

6. Computational details 18

Literature 23

1. General Considerations.

All manipulations were carried out under dinitrogen or argon atmosphere using Schlenk techniques or in glove boxes under atmospheres maintained below 1 ppm of O_2 and H_2O. Glassware was heated under vacuum for approx. 10 min using a heat gun at 650 °C prior to use. Solvents were used purified from an MBraun solvent purification system (SPS) and stored over potassium or molecular sieves. C_6D_6 was stored in a glove box and dried over 3 Å molecular sieves. IR spectra were recorded on a Bruker ALPHA spectrometer with an ATR sampling unit. Raman measurements were performed at a triple Raman spectrometer (Acton TriVista TR_{557}) employing an Ar^+ ion laser with a wavelength of 488 nm and a power of 100 mW. Elemental analyses were performed with a HEKA Euro 3000 elemental analyser. NMR spectra were recorded with Avance III 500 NMR spectrometer at room temperature. Effective magnetic moments were determined by the Evans NMR method using the shift of the tetramethyl silane (TMS) resonance.[1] Simple diamagnetic correction was applied.[2]

All materials were obtained from commercial vendors as ACS reagent-grade or better and used as received, if not stated otherwise. The reagents $^{TCHP}NONH_2$,[3] $^{Trip}NONH_2$[4] and 5% w/w K/KI[5] were prepared according to literature procedures.

For most of the presented complexes carbon values, collected during elemental analysis, were reproducibly below the range usually considered as acceptable, although crystalline material was analysed. The presented complexes are highly sensitive and quickly undergo decomposition, which may impede appropriate data collection. We also refer to a recent study that is questioning the reasonableness of narrow deviation guidelines for elemental analysis.[6]

For compounds **1-5** ^1H NMR spectra did not display any detectable signal in the region of 200 ppm to –200 ppm. Therefore, no NMR data is reported for all manganese compounds.

2. Synthetic procedures

2.1 Synthesis of [(^{Trip}NON)Mn(THF)$_2$], 1

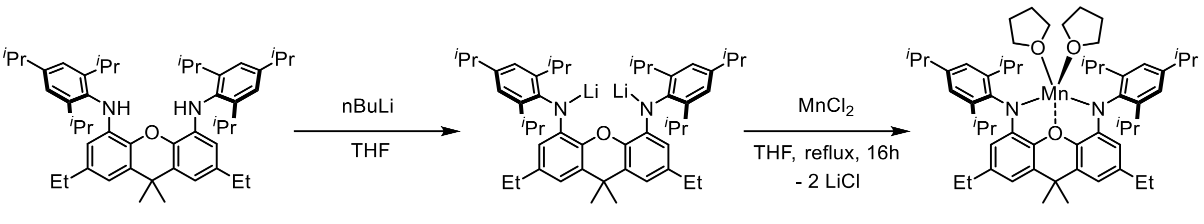

(^{TRIP}NON)Li$_2$ 1

nBuLi (0.8 mL, 1.8 mmol, 2.5 M in hexane) was added dropwise to a THF solution (15 mL) of ^{Trip}NONH$_2$ (550 mg, 0.78 mmol). After stirring for 1 h MnCl$_2$ (150 mg, 1.20 mmol) was added and the mixture was refluxed overnight. The solvent was removed and the residue extracted with toluene (15 mL). The solvent was again removed under reduced pressure and the solid residue was washed with pentane (3x 2 mL). After drying [(^{Trip}NON)Mn(THF)$_2$], **1**, was obtained as colourless solid (384 mg, 0.43 mmol, 55%).

Crystals suitable for X-ray diffraction analysis were grown via slow evaporation of a hexane solution.

μ_{eff} (C_6D_6, Evans method, 298 K): 5.80 μ_B

μ_{eff} (solid, SQUID, 298 K): 5.97 μ_B

ATR-IR (solid obtained from C_6D_6 solution): ν (cm^{-1}) = 2957 (s), 2927 (m), 2865 (w), 2280 (w, C_6D_6), 1614 (w), 1578 (w), 1479 (m), 1442 (s), 1346 (m), 1300 (m), 1261 (w), 1218 (m), 1192 (m), 1100 (w), 1092 (m), 1027 (s), 877 (m), 849 (m), 811 (s) 636 (w).

Elemental analysis calc. (%) for $C_{57}H_{82}MnN_2O_3$ (898.23 g*mol^{-1}): C 76.22, H 9.20, N 3.12; found: C 75.74, H 9.40, N 3.16.

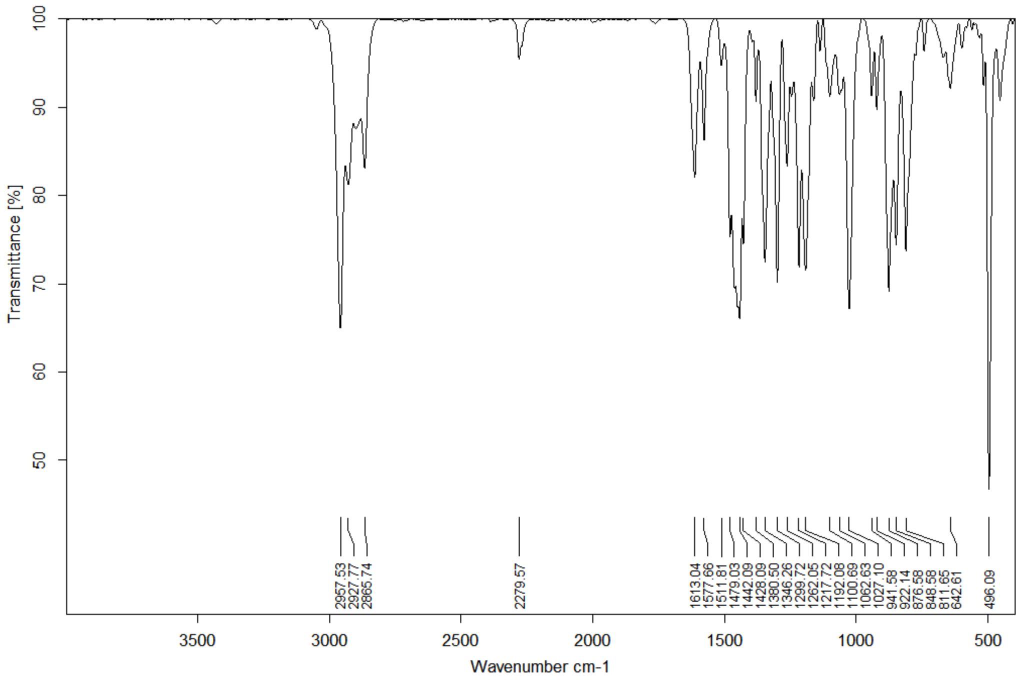

Figure S1: ATR-IR spectrum of **1** (solid obtained from C_6D_6 solution).

2.2 Synthesis of [(^{TCHP}NON)MnTHF], 2

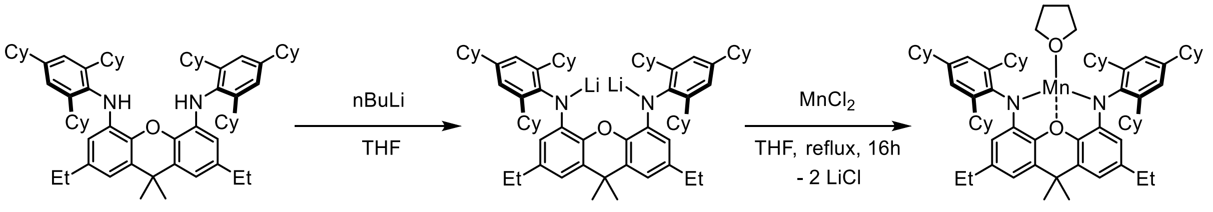

(^{TCHP}NON)Li_2 **2**

nBuLi (0.6 mL, 1.30 mmol, 2.5 M in hexane) was added dropwise to a THF solution (15 mL) of ^{TCHP}NON-H_2 (550 mg, 0.59 mmol). After stirring for 1 h $MnCl_2$ (150 mg, 1.20 mmol) was added and the mixture was refluxed overnight. The solvent was removed and the residue extracted with toluene (15 mL). The solvent was again removed under reduced pressure and the solid residue was washed with pentane (3x 2 mL). After drying [(^{TCHP}NON)MnTHF], **2**, was obtained as yellow solid (462 mg, 0.43 mmol, 72%).

Crystals suitable for X-ray diffraction analysis were grown via slow evaporation of a hexane solution.

μ_{eff} (C_6D_6, Evans method, 298 K): 5.34 μ_B

μ_{eff} (solid, SQUID, 298 K): 5.76 μ_B

ATR-IR (solid obtained from C_6D_6 solution): ν (cm^{-1}) = 2960 (w), 2921 (m), 2849 (m), 2280 (w, C_6D_6), 1617 (w), 1581 (w), 1478 (m), 1447 (m), 1349 (w), 1298 (w), 1260 (m), 1211 (w), 1091 (m), 1051 (s), 862 (m), 798 (w).

Elemental analysis calc. (%) for $C_{71}H_{98}MnN_2O_2$ (1066.52 g*mol^{-1}): C 79.96, H 9.26, N 2.63; found: C 78.67, H 9.58, N 2.64

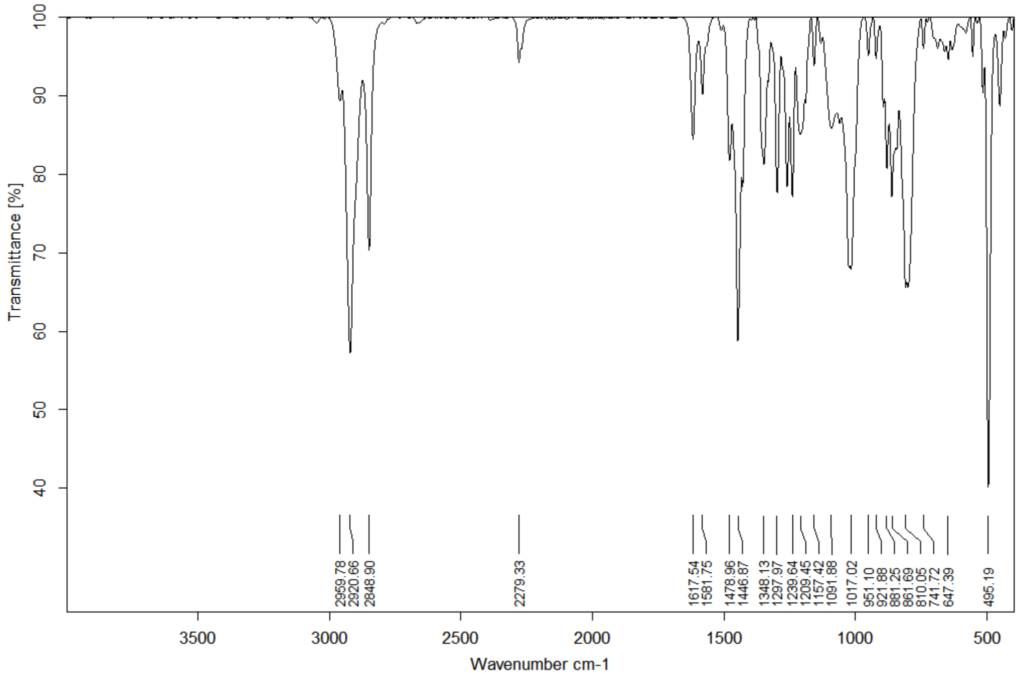

Figure S2: ATR-IR spectrum of **2** (solid obtained from C_6D_6 solution).

2.3 Synthesis of [(K(^{Trip}NON)Mn)$_2$], 3

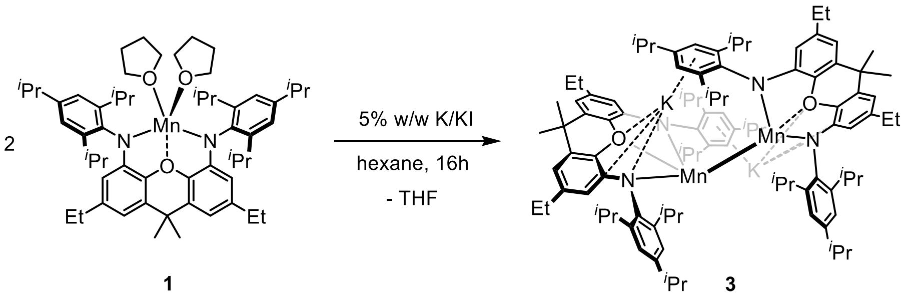

A mixture of **1** (500 mg, 0.57 mmol) and 5% w/w K/KI (700 mg, 0.90 mmol) was suspended in hexane in an N_2 atmosphere and stirred for 16 h. During this time the colour of the solution changed from yellow to orange red. After evaporation of the solvent the residue was extracted with toluene (2x 15 mL). The organic phase was dried under vacuum and the residue was dissolved in a minimum of hexane. The solution was kept at room temperature for 2 h and then stored at –30 °C to afford [(K(^{Trip}NON)Mn)$_2$], **3**, as red crystalline material. The mother liquor was concentrated and stored at -30 °C yielding a second crop of crystals (combined yield: 127 mg, 0.08 mmol, 28 %)

Single crystals suitable for X-ray diffraction analysis were grown from a saturated solution of hexane at –30 °C.

μ_{eff} (C_6D_6, Evans method, 298 K): 7.43 μ_B

μ_{eff} (solid, SQUID, 298 K): 7.98 μ_B

ATR-IR (solid obtained from C_6D_6 solution): ν (cm^{-1}) = 2959 (s), 2927 (w), 2866 (w), 2280 (w, C_6D_6), 1611 (m), 1577 (w), 1512 (w), 1463 (s), 1440 (s), 1352 (m), 1302 (m), 1260 (m), 1218 (m), 1182 (m), 1097 (w), 1062 (w), 1027 (s), 878 (m), 845 (m), 810 (s), 646 (w).

Elemental analysis calc. (%) for $C_{98}H_{132}K_2Mn_2N_4O_2$ (1586.23 g*mol^{-1}): C 74.21, H 8.39, N 3.53; found: C 72.62, H 8.63, N 3.50.

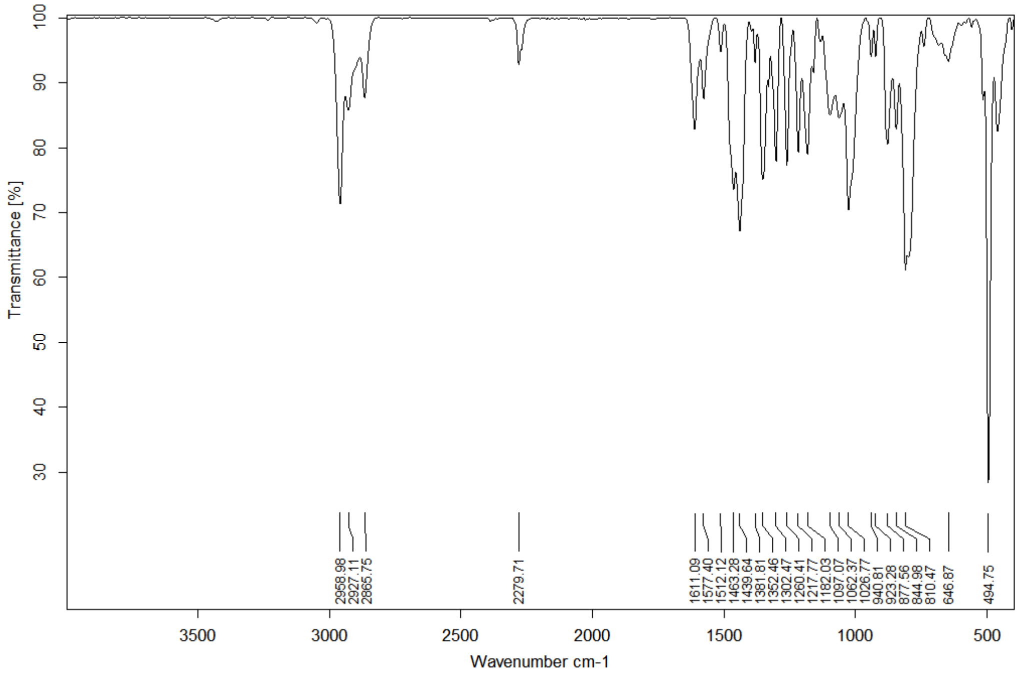

Figure S3: ATR-IR spectrum of **3** (solid obtained from C_6D_6 solution).

2.4 Synthesis of [(K(^{TCHP}NON)Mn)$_2$(μ-η^1:η^1-N$_2$)], 4

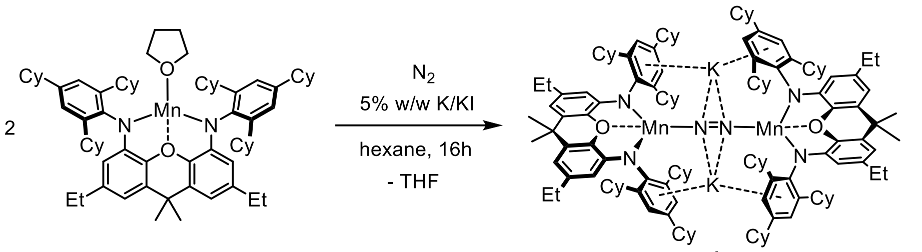

2 **4**

A mixture of **2** (500 mg, 0.47 mmol) and 5% w/w K/KI (550 mg, 0.70 mmol) was suspended in hexane in an N_2 atmosphere and stirred for 16 h. During this time the colour of the solution changed from yellow to orange red. After evaporation of the solvent the residue was extracted with toluene (2x 15 mL). The organic phase was dried under vacuum and the residue was dissolved in a minimum of hexane. The solution was kept at room temperature for 2 h and then stored at –30 °C to afford [(K(^{TCHP}NON)Mn)$_2$(μ-η^1:η^1-N$_2$)], **4**, as an orange crystalline material. The mother liquor was concentrated and stored at –30 °C yielding a second crop of crystals (combined yield: 183 mg, 0.09 mmol, 36 %).

Single crystals suitable for X-ray diffraction analysis were grown from saturated solution of benzene.

μ_{eff} (C_6D_6, Evans method, 298 K) = 8.21 μ_B.

μ_{eff} (solid, SQUID, 298 K) = 8.48 μ_B.

ATR-IR (solid obtained from C_6D_6 solution): ν (cm^{-1}) = 2959 (w), 2922 (s), 2849 (m), 2270 (w, C_6D_6), 1610 (w), 1580 (w), 1511 (w), 1479 (w), 1442 (s), 1348 (w), 1302 (w), 1273 (w), 1239 (w), 1213 (w), 1189 (m), 1093 (w), 1025 (m), 871 (w), 811 (m), 646 (w).

Raman (N-N str.): ν = 1615 cm^{-1}.

Elemental analysis calc. (%) for $C_{134}H_{180}K_2Mn_2N_6O_2$ (2095.03 g*mol^{-1}): C 76.82, H 8.66, N 3.73; found: C 74.67, H 8.76, N 3.56.

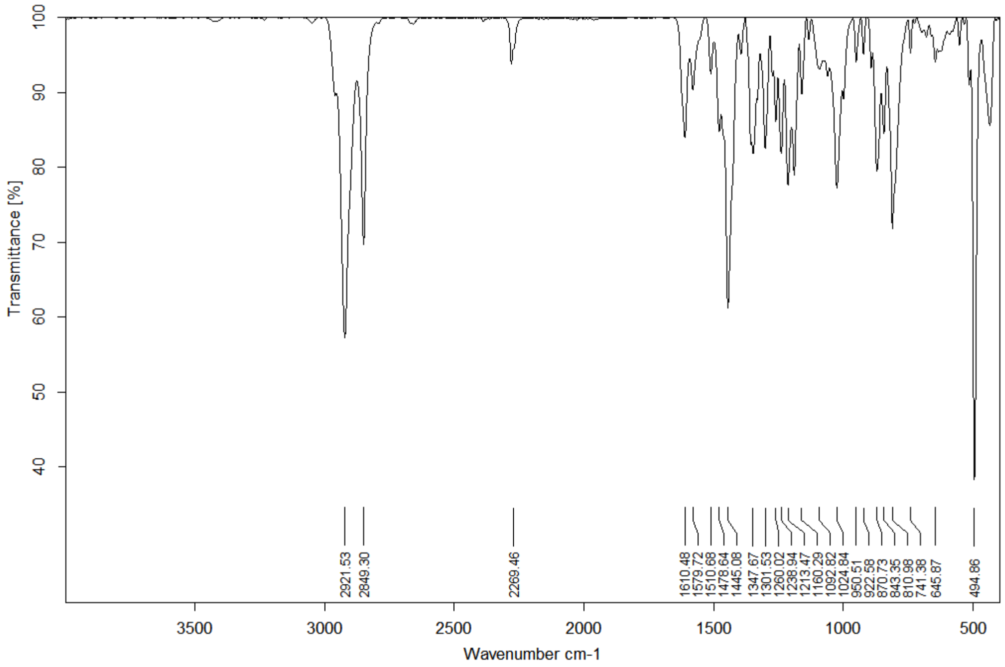

Figure S4: ATR-IR spectrum of **4** (solid obtained from C_6D_6 solution).

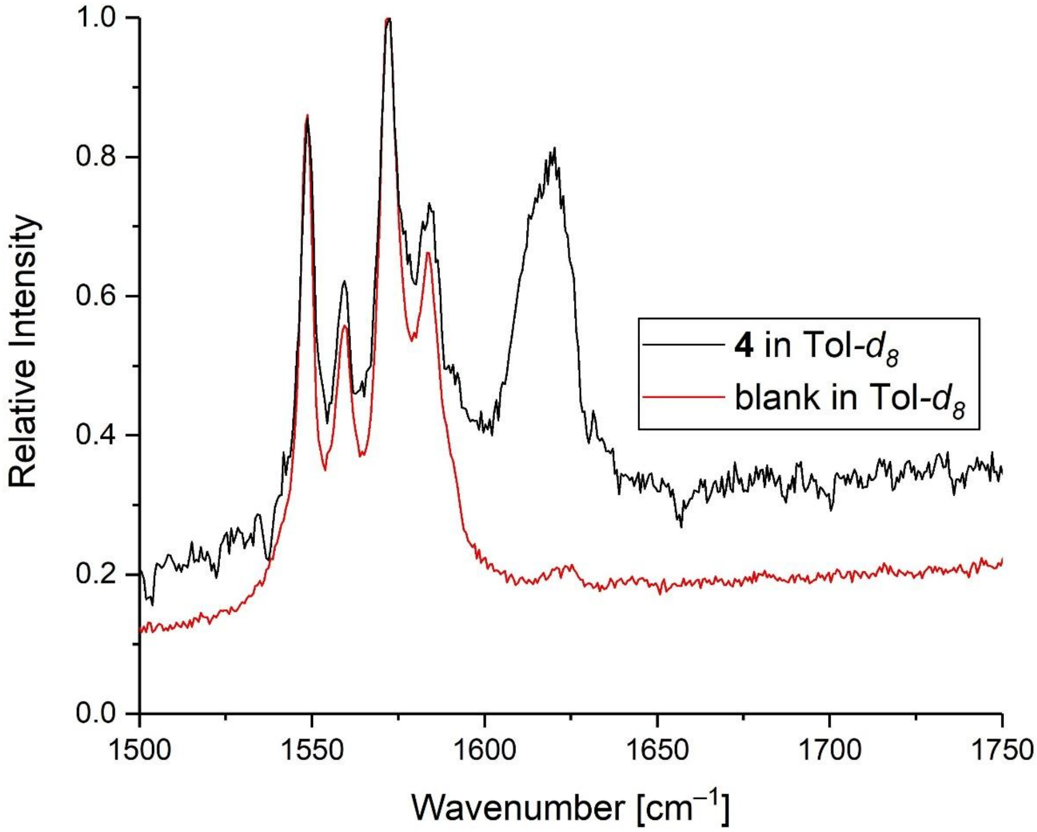

Figure S4: Overlay of the Raman spectra of **4** dissolved in toluene-d_8 (black trace) with the N-N stretching band labelled in comparison to a corresponding measurement of neat toluene-d_8 (red trace).

2.5 Synthesis of [(^{TCHP}NON)Mn(C_6H_5)], 5

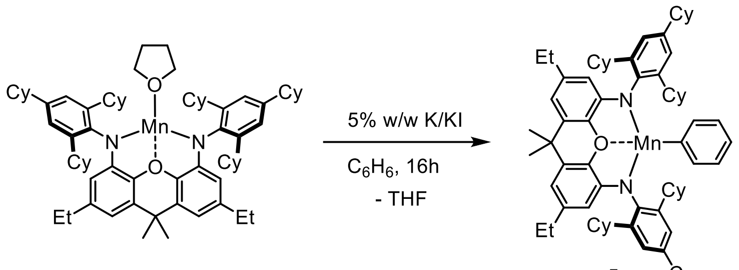

A mixture of **2** (500 mg, 0.47 mmol) and 5% w/w K/KI (550 mg, 0.70 mmol) was suspended in benzene in an argon atmosphere and stirred for 16 h. During this time the colour of the solution changed from yellow to deep red. After evaporation of the solvent the residue was extracted with hexane (2x 25 mL). The organic phase was concentrated under vacuum to about 5 mL and then stored at –30 °C to afford [(^{TCHP}NON)Mn(C_6H_5)], **5**, as red crystalline material. The mother liquor was concentrated and stored at –30 °C yielding a second crop of crystals (combined yield: 214 mg, 0.20 mmol, 42 %).

Single crystals suitable for X-ray diffraction analysis were grown from saturated solution of benzene.

μ_{eff} (C_6D_6, Evans method, 298 K) = 3.82 μ_B

μ_{eff} (solid, SQUID, 298 K) = 4.31 μ_B

ATR-IR (solid obtained from C_6D_6 solution): ν (cm^{-1}) = 2961 (w), 2921 (s), 2849 (m), 2280 (w, C_6D_6), 1623 (w), 1580 (w), 1510 (w), 1480 (w), 1442 (s), 1347 (w), 1302 (w), 1240 (w), 1214 (w), 1156 (m), 1091 (w), 1026 (m), 9951 (w), 800 (m), 686 (w).

Elemental analysis calc. (%) for $C_{73}H_{95}MnN_2O$ (1074.70 g*mol^{-1}): C 81.83, H 8.94, N 2.61; found: C 78.85, H 9.13, N 2.12.

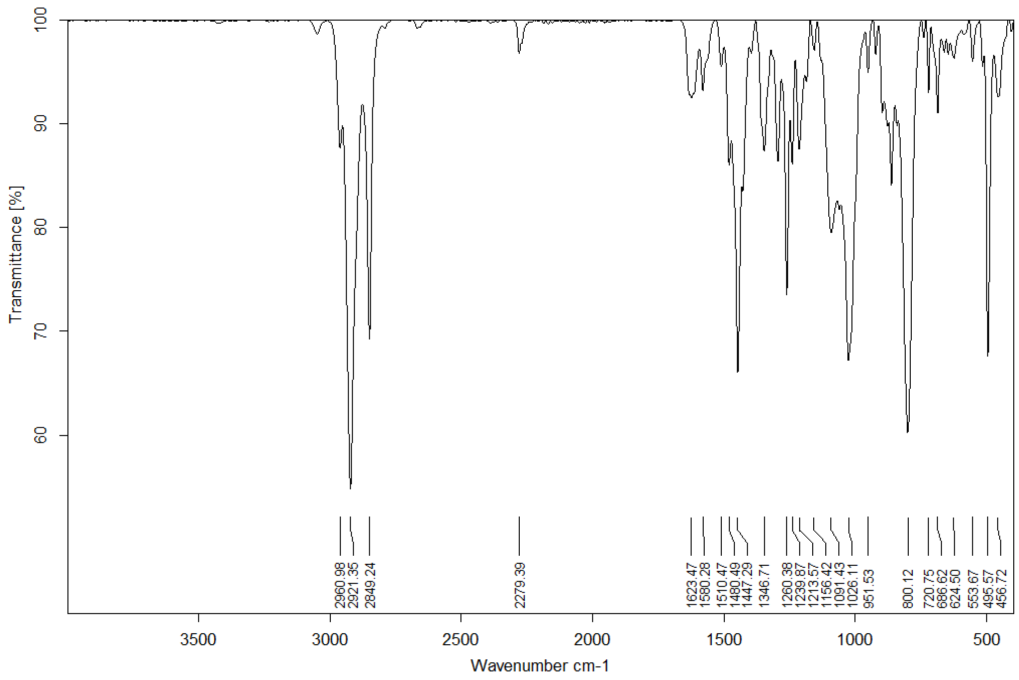

Figure S6: ATR-IR spectrum of **5** (solid obtained from C_6D_6 solution).

3. SQUID Measurements

Magnetic measurements were performed with a QuantumDesign MPMS3 SQUID magnetometer. The samples were prepared in a glove box in VSM powder capsules which were sealed with a piece of Teflon tape. Both the capsules and the Teflon tape were dried in a Schlenk flask under vacuum at 110 °C for five days. A brass sample holder was used. The measurement was carried out in VSM mode from 2 K to 300 K in a magnetic field of 7 T after cooling down at zero field (**2**, **5**) or in a field of 7 T (**1**, **3**, **4**). A background correction was applied by subtracting the magnetic moments of an empty capsule sealed with a piece of Teflon tape using the same measurement sequence as for the sample. A diamagnetic correction was performed using Pascal's constants.[2] The simulation of the experimental magnetic data was performed with N. F. Chilton's PHI software (version 3.1.6)[7] using the following spin Hamiltonian:

$$\hat{H} = \mu_B \sum_{i=1}^{N} g * \hat{S}_i * B_0 - 2 \sum_{i \neq j}^{i,j \in N} \hat{S}_i * J_{ij} * \hat{S}_j \text{ for } \mathbf{3}$$

Simulation results for **3**:

g	error(g)	J_{xx} [cm^{-1}]	error(J_{xx})	J_{yy} [cm^{-1}]	error(J_{yy})	J_{zz} [cm^{-1}]	error(J_{zz})	D [cm^{-1}]	error(D)
1.987	0.0014	2.15	0.09	-1.85	0.08	-8.68	0.14	13.84	0.32

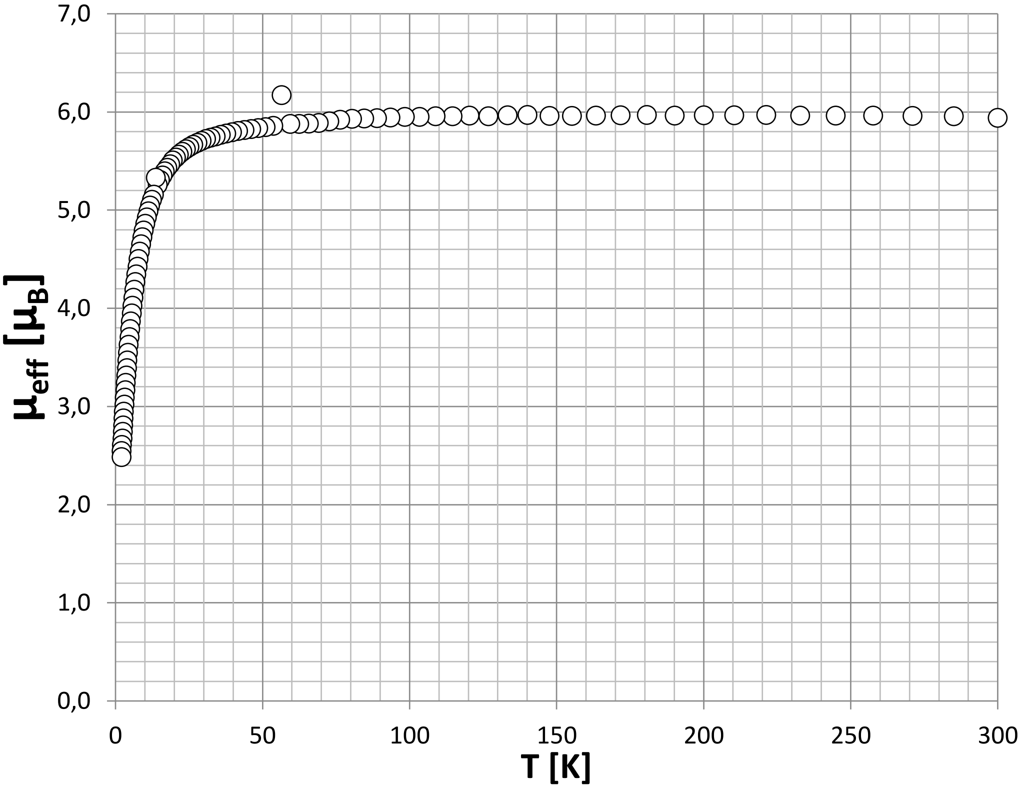

Figure S7: Effective magnetic moment of **1** at variable temperature.

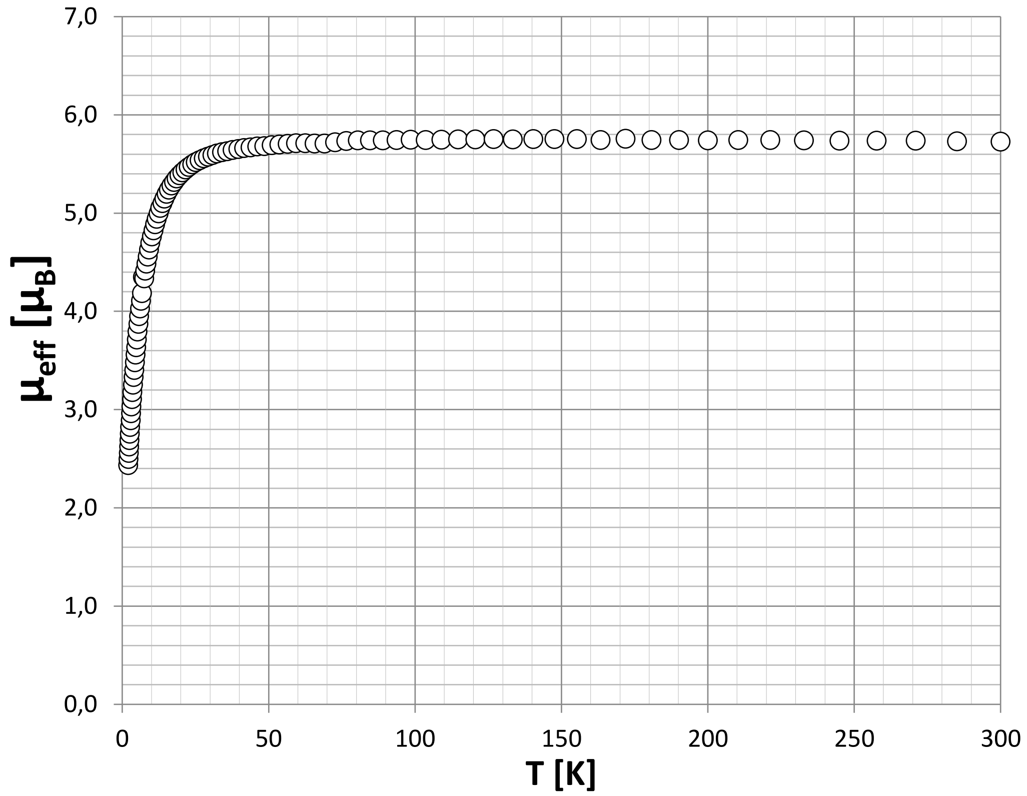

Figure S8: Effective magnetic moment of **2** at variable temperature.

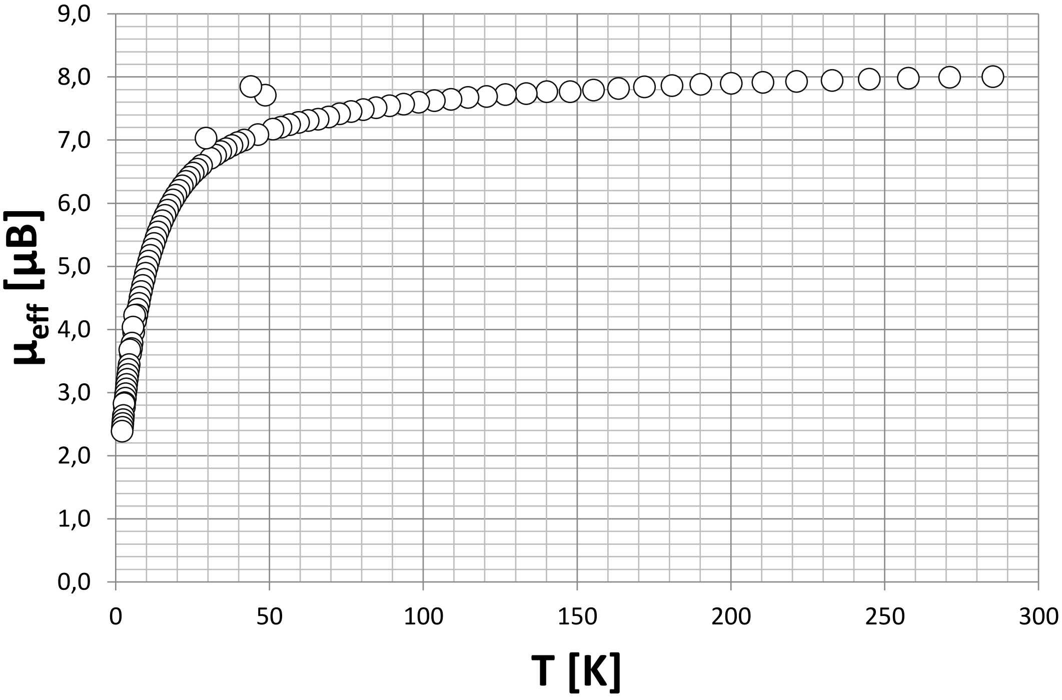

Figure S9: Effective magnetic moment of **3** at variable temperature.

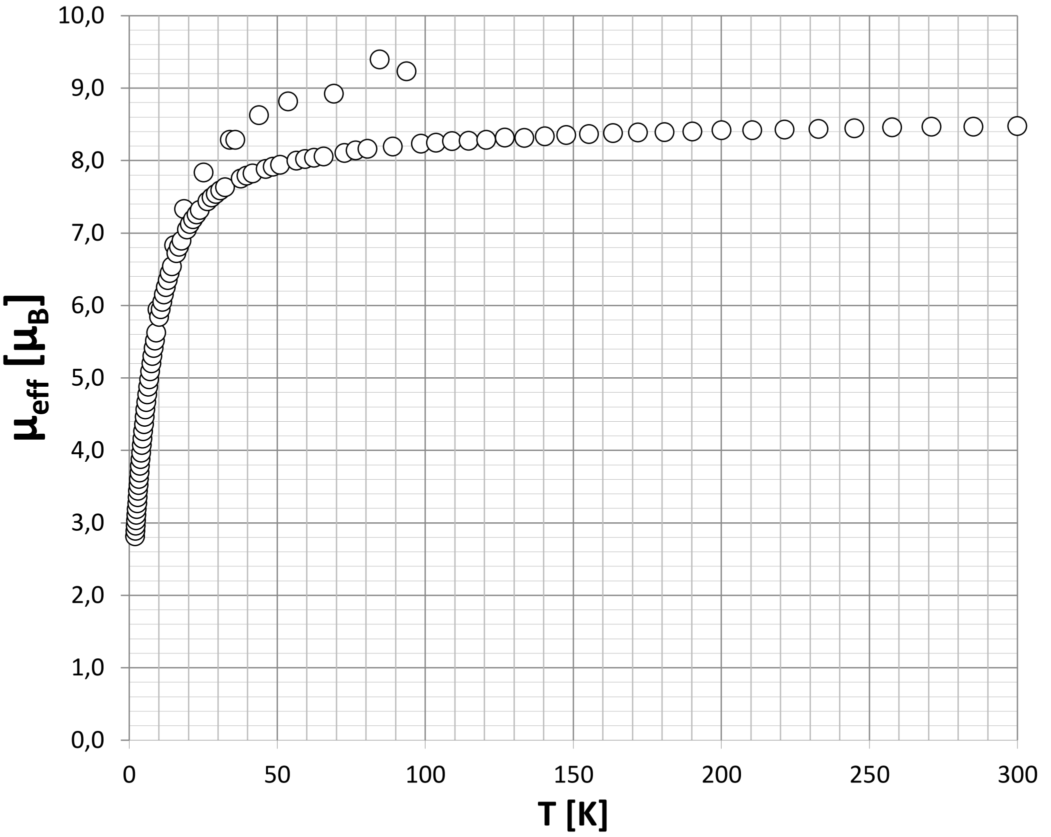

Figure S10: Effective magnetic moment of **4** at variable temperature.

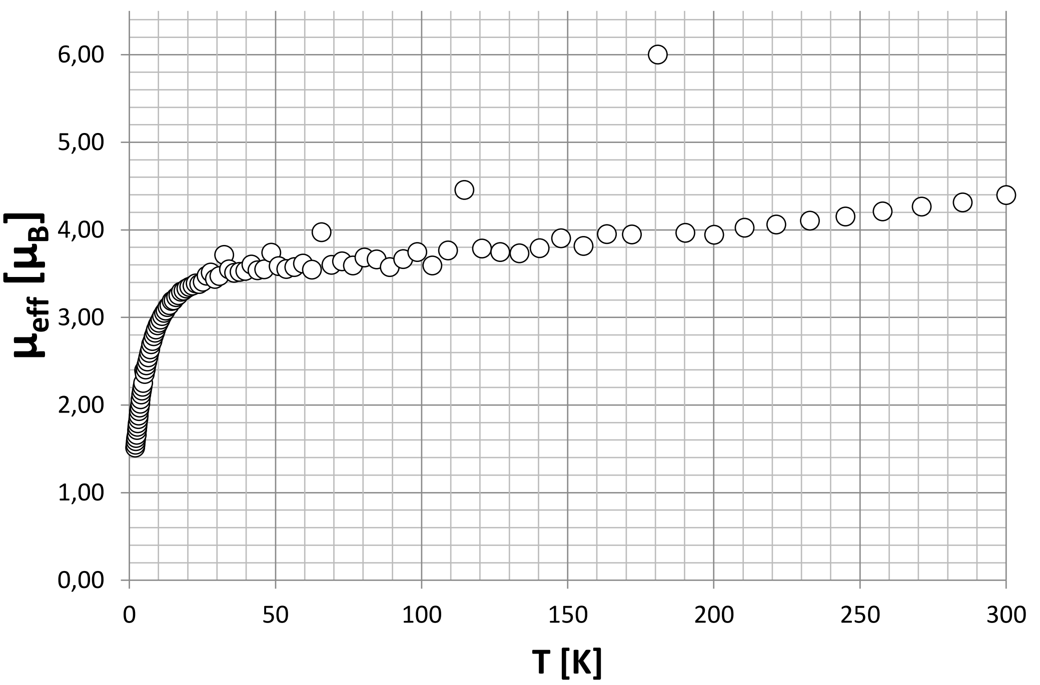

Figure S11: Effective magnetic moment of **5** at variable temperature.

4. EPR Measurements

X-band CW EPR spectra used for qualitative analysis were collected by a Bruker EMXplus Instrument at a frequency of ca. 9.35 GHz (X-Band) in perpendicular mode, equipped with the Bruker ER4119-HS probehead. All samples were measured at an average temperature of 13 K as frozen solutions, using a liquid helium recirculating cooling system provided by ColdEdge.

1 exhibits a high-spin signal (g value of approximately 4.3), which corroborates the SQUID and NMR measurements. Mn^{II} has a nuclear spin (I) of 5/2 and therefore exhibits a hyperfine splitting of six lines. The multi-line hyperfine observed in the referred signal shows the typical splitting for such a system, with the addition of narrower lines that are, most likely, due to the hyperfine interaction with the neighbouring nitrogen (I = 1, splitting of 3 lines) atoms in the ligand. In the high-field region, at g=2.0, a narrow signal with a three-line shape appears, which is characteristic of a nitrogen-based organic radical. Quantification of this species compared to a $CuSO_4$ standard shows it as an impurity (>1%).

2 exhibits a very similar EPR signal compared to **1**, with a g value of approximately 4.3. Again, the main signal exhibits the fingerprint hyperfine splitting of Mn(II), with additional narrower lines. The intensity of the signal is higher, due to the higher concentration, but the overall shape is very similar to the EPR of **1**. Similarly to **1**, multi-line hyperfine splitting is observed, which most probably arises from the interaction with the N atoms around. In this case, the signal at g=2.0 appears smaller, and quantification compared to a $CuSO_4$ standard shows it as an impurity (>1%).

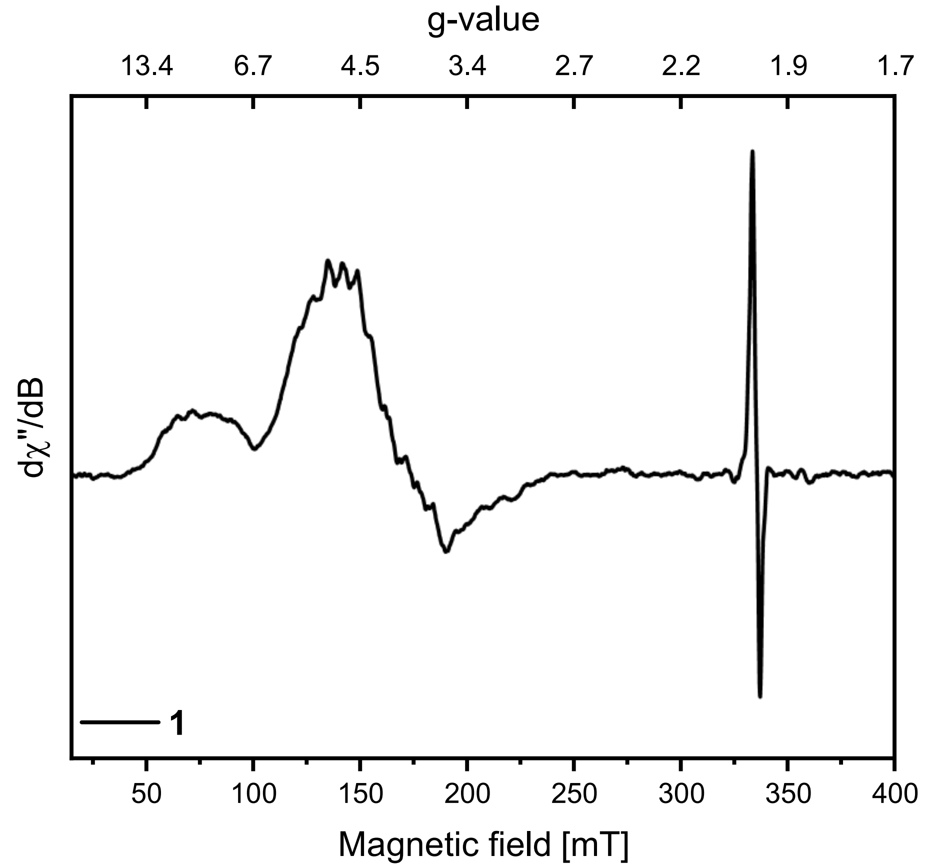

Figure S12: CW X-Band EPR spectrum of **1** (1 mM) recorded at 13 K in toluene (microwave power 0.06 mW, modulation amplitude 5 G).

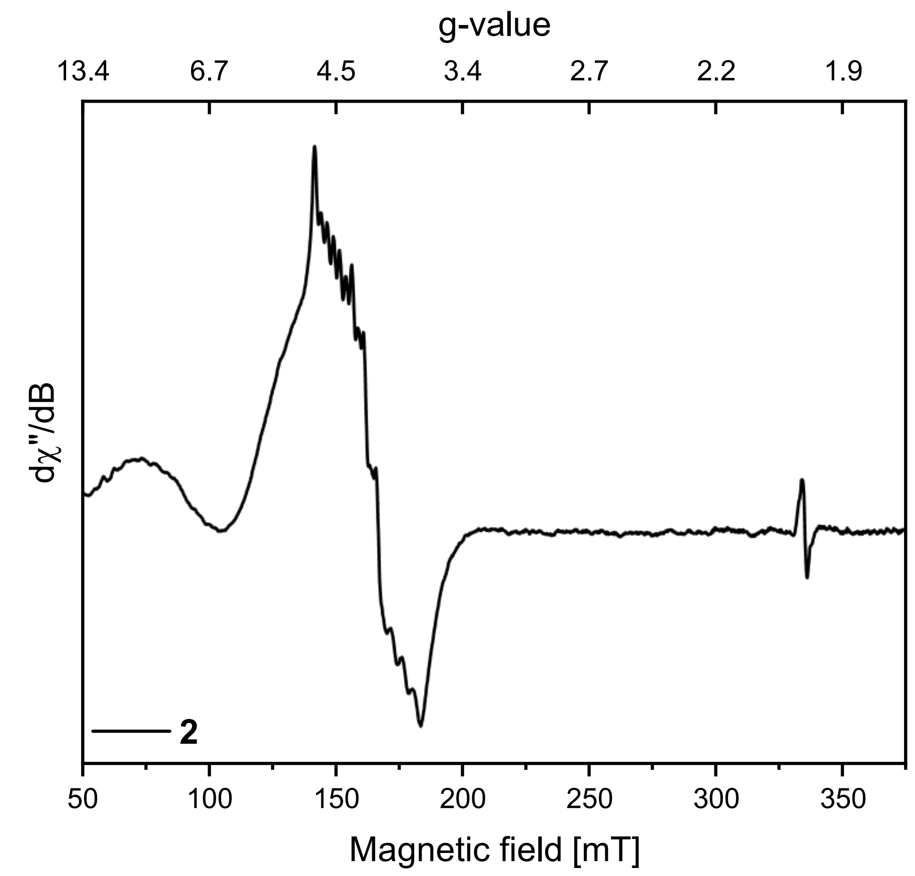

Figure S13: CW X-Band EPR spectrum of **2** (2 mM) recorded at 13 K in toluene (microwave power 0.06 mW, modulation amplitude 5 G).

5. Crystallographic Data

Crystals suitable for X-ray structural determination were mounted in silicone oil. Crystallographic measurements were made using either a Rigaku Xtalab Synergy Dualflex diffractometer with a graphite monochromator with Cu Kα radiation (1.54180 Å), a BRUKER D8 VENTURE area detector with Mo-Kα radiation (λ = 0.71073 Å) or the MX1 beamline of the Australian Synchrotron (λ = 0.71090 Å). The software package Blu-Ice[8] was used for synchrotron data acquisition, while the program XDS[9] was employed for synchrotron data reduction. The structures were solved by intrinsic phasing method (SHELXT-2013[10]) and refined by full matrix least square procedures based on F2 with all measured reflections (SHELXT-2014[11] in the graphical user interface SHELXle[12]) with anisotropic temperature factors for all non-hydrogen atoms. Squeeze[13] refinement have been applied to the data of complex **3**.

CCDC numbers 2439625 - 2439629 contain the supplementary crystallographic data for this paper. These data can be obtained free of charge from the Cambridge Crystallographic Data Centre via www.ccdc.cam.ac.uk/data_request/cif.

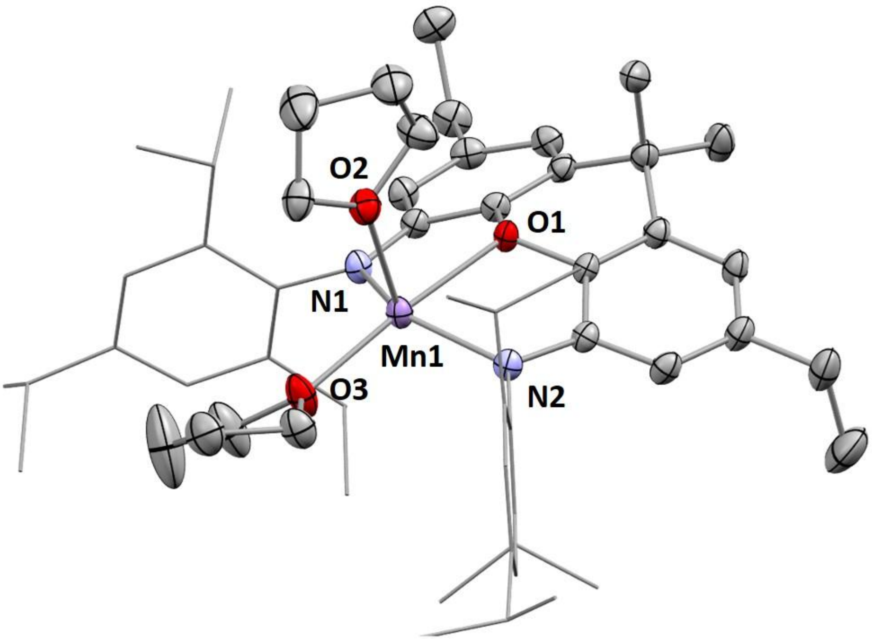

Figure S14: Molecular structure of **1** as determined by X-ray diffraction analysis. Thermal ellipsoids are shown at the 50% probability level. H atoms are omitted for clarity. Selected bond lengths (Å) and angles (°): Mn1-O1 2.248(1), Mn1-O2 2.242(1), Mn1-O3 2.188(1), Mn1-N1 2.105(1), Mn1-N2 2.097(2), N1-Mn1-N2 138.25(6), O1-Mn1-O2 95.66(5), O2-Mn1-O3 86.52(5), O1-Mn1-O3 175.93(5). Geometry index: τ^5= 0.63.

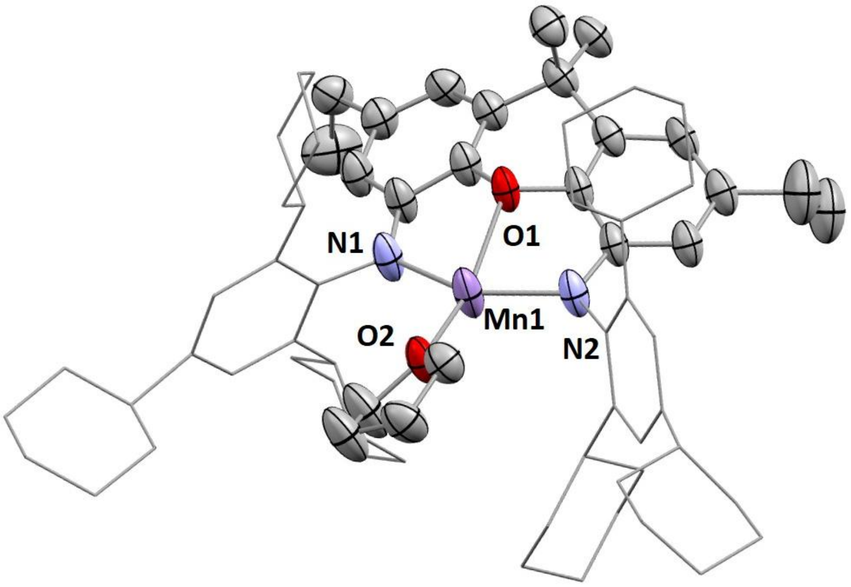

Figure S15: Molecular structure of **2** as determined by X-ray diffraction analysis. Thermal ellipsoids are shown at the 50% probability level. H atoms are omitted for clarity. Selected bond lengths (Å) and angles (°): Mn1-O1 2.226(3), Mn1-O2 2.16(1), Mn1-N1 2.038(3), Mn1-N2 2.033(4), O1-Mn1-O2 150.4(4), N1-Mn1-N2 146.06(1). Geometry index: τ^4= 0.43, $\tau^{4'}$ = 0.41.

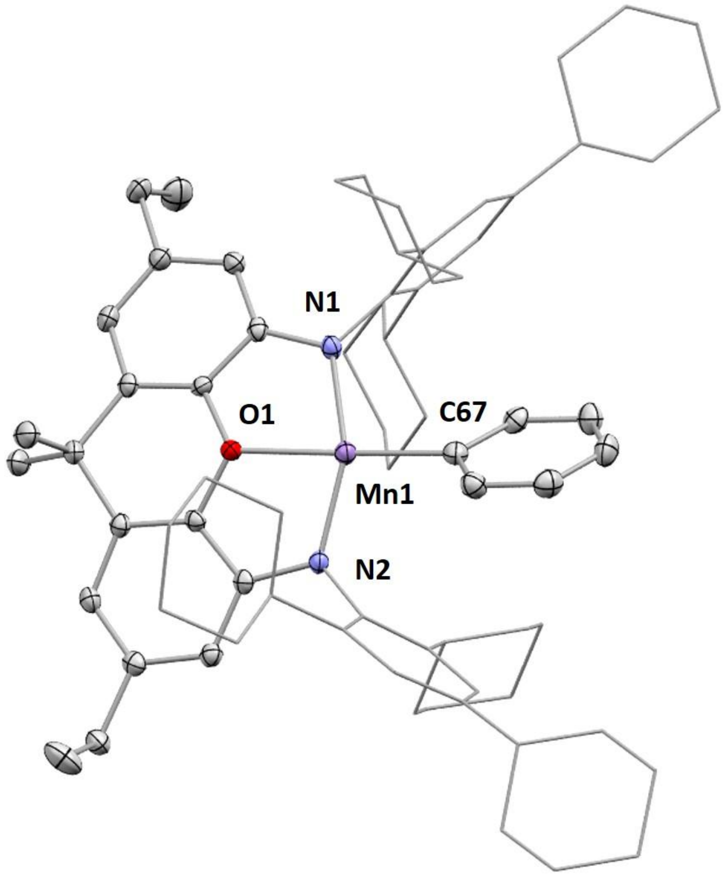

Figure S16: Molecular structure **5** as determined by X-ray diffraction analysis. Thermal ellipsoids are shown at the 50% probability level. H atoms and co-crystallised solvent molecules are omitted for clarity. Selected bond lengths (Å) and angles (°): Mn1-O1 2.015(2), Mn1-N1 1.972(1), Mn1-N2 1.964(2), Mn1-C67 2.025(2), O1-Mn1-C67 170.12(7), N1-Mn1-N2 154.00(7). Geometry index: τ^4= 0.25, $\tau^{4'}$ = 0.20.

Table S1: Crystallographic Data for compounds **1**-**3**.

**Crystal structure**	**[(^{TRIP}NON)Mn(THF)$_2$], 1**	**[(^{TCHP}NON)MnTHF], 2**	**[(K(^{TCHP}NON)Mn)$_2$], 3**
CCDC Depositon Nr	2439625	2439626	2439627
Chemical formula	$C_{57}H_{82}MnN_2O_3$	$C_{71}H_{98}MnN_2O_2$	$C_{98}H_{132}K_2Mn_2N_4O_2$
M_r/g*mol^{-1}	898.18	1066.45	1586.15
Temperature/K	123	100	100 K
Wave length/Å	1.54184	0.71073	0.71073
Crystal system	monoclinic	orthorhombic	monoclinic
Space group	$P2_1/c$	$P2_12_12_1$	$C2/c$
a/Å	26.9491(2)	13.180(3)	25.350 (2)
b/Å	19.2278(1)	20.720(4)	28.751 (2)
c/Å	21.1237(2)	23.230(5)	17.2873 (15)
α/°	90	90	90
β/°	109.005(1)	90	112.275 (3)
γ/°	21.1237(2)	90	90
Cell volume/ Å^3	10349.05(15)	6344(2)	11659.1 (18)
Z	8	4	4
P_{calc}/g*cm^{-1}	1.153	1.117	0.904
μ / mm^{-1}	2.399	0.25	0.326
F(0 0 0)	3896.0	2316.0	3408.0
Crystal size/mm	0.37x0.25x0.19	0.05x0.03x0.02	0.14×0.14×0.10
2θ area/°	4.0 - 80.4	1.3 – 26.4	1.9 – 26.6
Measured reflexes	110984	155700	322786
Independent reflexes	22101	12966	12052
Parameters/ restrains	1178/0	1021/5646	529/554
R_{int}	0.047	0.044	0.107
R_1 (I>2σ(I))	0.044	0.060	0.059
wR_2 (all data)	0.117	0.176	0.175
GooF (all data)	1.048	1.039	1.07
Flack-parameter	-	0.03(2)	-
Max. u. min $\Delta\rho_{elect.}$ /e Å^{-3}	0.53/ −0.68	0.60/ −0.64	1.26/ −0.55
Absorption correction	Multi-scan	Multi-scan/XDS	Multi-Scan
Max/min. transmission	0.625/1.000	Not reported by XDS	0.745/ 0.681

Table S2: Crystallographic Data for compounds **4** and **5**.

**Crystal structure**	**[(K$_2$(^{TCHP}NON)Mn)$_2$N$_2$], 4**	**[(^{TCHP}NON)Mn(Ph)], 5**	
CCDC Depositon Nr	2439628	2439629	
Chemical formula	$C_{134}H_{180}K_2Mn_2N_6O_2$ + 2.5 C_6H_6	$C_{73}H_{95}MnN_2O$ + 3 C_6H_6	
M_r/g*mol^{-1}	2290.18	1305.77	
Temperature/K	123	101	
Wave length/Å	1.54184	0.71073	
Crystal system	triclinic	triclinic	
Space group	*P*-1	*P*-1	
a/Å	16.7043(4)	11.9443(18)	
b/Å	17.7723(4)	14.1570(17)	
c/Å	23.4821(3)	23.017(3)	
α/°	80.762(1)	79.902(5)	
β/°	88.284(2)	80.829(5)	
γ/°	72.195(2)	81.288(5)	
Cell volume/ Å^3	6549.9(2)	3752.2(9)	
Z	2	2	
P_{calc}/g*cm^{-1}	1.161	1.156	
µ / mm^{-1}	2.540	0.224	
F(0 0 0)	2470.0	1412.0	
Crystal size/mm	0.19x0.11x0.09	0.19×0.12×0.05	
2θ area/°	3.5 – 81.2	1.8 -26.4	
Measured reflexes	130035	119312	
Independent reflexes	27846	15423	
Parameters/ restrains	1688/ 4606	915/1275	
R_{int}	0.150	0.095	
R_1 (I>2σ(I))	0.105	0.047	
wR_2 (all data)	0.283	0.120	
GooF (all data)	1.037	1.02	
Flack-parameter	-	-	
Max. u. min $\Delta\rho_{elect.}$ /e Å^{-3}	1.64/ −1.00	0.37/ −0.65	
Absorption correction	Multi-scan	Multi-scan	
Max/min. transmission	0.634/1.000	0.682/0.745	

6. Computational details

The DFT calculations were carried out by employing hybrid functional (B3PW91)[14] along with small core pseudopotential Stuttgart basis set[15] for manganese and Pople basis set[16] (6-311G** for K atoms and 6-31G** for the rest of the atoms). Dispersion corrections were included in our calculations by employing D3 version of Grimme's dispersion with Becke-Johnson damping.[17] Frequency calculations were performed to locate minima for the optimised structures and for obtaining thermal corrections over the energies. All the calculations were performed using Gaussian 09 suite of programs.[18]

On this level, DFT calculations were carried out on complex **3** to try to explain the bonding situation as well as the peculiar $(Mn^{I})_2$ electronic configuration. Different spin states were considered in the calculations namely an open-shell singlet, a septet (S=3) and a nonet (S=4), which correspond to different spin arrangements of Mn^{I} centres (d^6 or s^1d^5) valence configuration. Quite unexpectedly with respect to the magnetic measurement, the open-shell singlet (5 unpaired electrons per Mn, strongly antiferromagnetically coupled) is far lower than the two other spin states, with the septet being the highest in energy (more than 55 kcal/mol above) while the nonet is 25 kcal/mol higher in energy than the open-shell singlet. The optimised geometry for the open-shell singlet compares well with the experimental one (see Table S5). For instance, the Mn-Mn distance is satisfactorily reproduced (2.89 Å vs. 2.95 Å experimentally) and all the Mn-O and Mn-N distances are reproduced within 0.03 Å. The bonding analysis carried out using both molecular orbitals (MO) and Natural Bonding Orbital (NBO) analysis indicates the presence of a Mn-Mn single bond which is covalent (50-50 contribution of the two Mn) and formed with a *sd* hybrid atomic orbitals (60 to 80% s character). Scrutinizing the molecular orbital (MO) diagram, it appears that two sets of two singly occupied MOs (one α and one β) localized on each Mn centre are fully degenerated.

Computation of exchange coupling constant (*J*) between manganese centers in **3**:

Single point calculation for high-spin state (S=5) was computed starting from low spin (open shell singlet, S=0) optimized structure. *J* value was calculated using following equation:

$J=[E_{LS}-E_{HS}]/[2S_1S_2+S_2]$.

Spin configuration	E (Hartree)	Spin densities	
		Mn1	Mn121
HS, S=5	-5591.61528793	4.86	4.86
LS, open shell singlet, S=0	-5591.61939064	5.12	-5.12

The computed J value is -65.5 cm^{-1}.

Table S3: Energetics for different spin states computed for $[(K(^{Trip}NON)Mn)_2]$, **3**.

Spin states	ΔE (kcal/mol)
S=3	55.3
S=4	24.9
S=0 [open shell singlet]	0.0

Table S4: Selected structural parameters from DFT optimised geometry for **3.**

Atom labels	X-ray	DFT
		S=0
Mn1-O3	2.33	2.36
Mn1-N4	2.19	2.17
Mn1-N6	2.15	2.15
Mn121-O123	2.33	2.36
Mn121-N124	2.19	2.17
Mn121-N126	2.15	2.16
Mn1-Mn121	2.95	2.89

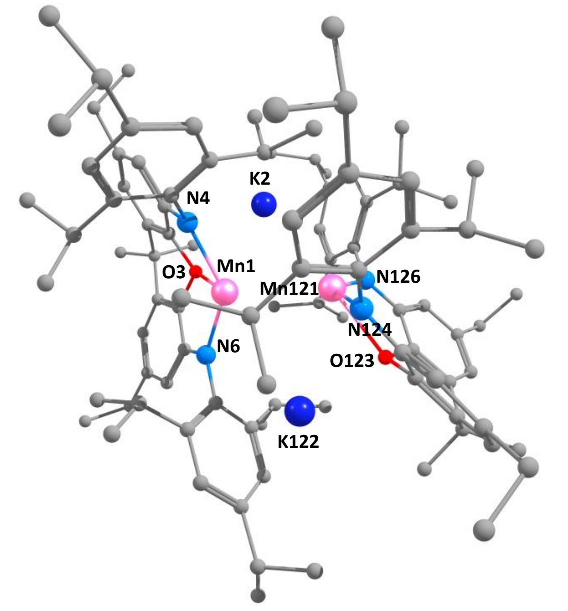

Table S5: DFT computed Wiberg bond index (WBI) between selected atoms in **3** (S=0).

Atom labels	WBI
Mn1-O3	0.0618
Mn1-N4	0.1660
Mn1-N6	0.1673
Mn121-O123	0.0623
Mn121-N124	0.1669
Mn121-N126	0.1675
Mn1-Mn121	0.7770

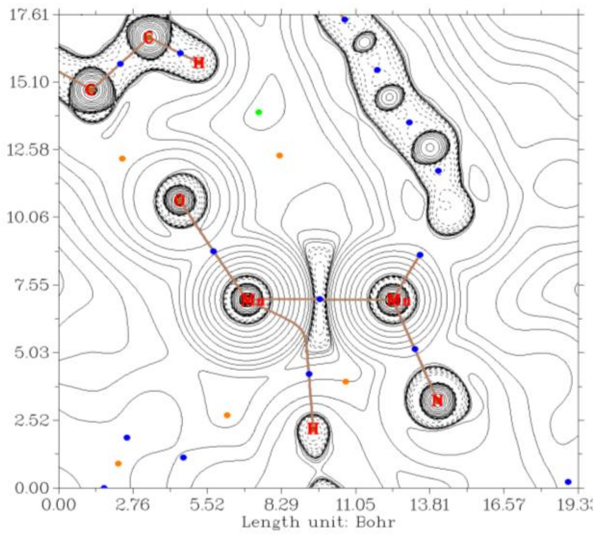

$\rho(r)$	$\nabla^2\rho(r)$	$G(r)$	$V(r)$	$H(r)$	ε
0.04	-0.01	0.007	-0.02	-0.01	0.11

Figure S17: Laplacian distribution for compound **3** (blue dots represent bond critical points, whereas orange dots represent ring critical points) and the computed BCP descriptors between the Mn centers. No non-nuclear attractor could be located between the two manganese centers.

Lone pairs of Mn atoms and bonding orbitals (Alpha Molecular Orbital, AMO) between selected atoms from NBO analysis in **3**:

(0.91648) BD (1) Mn1 -Mn121
(49.66%) 0.7047*Mn1 s (81.91%) p 0.19 (15.77%) d 0.03 (2.32%) f 0.00 (0.00%)
(50.34%) 0.7095*Mn121 s (60.33%) p 0.30 (18.25%) d 0.36 (21.42%) f 0.00 (0.00%)

(0.97663) LP (1) Mn1
s (0.12%) p 4.16 (0.49%) d 99.99 (99.39%) f 0.00 (0.00%)

(0.97329) LP (2) Mn1
s (0.01%) p 1.00 (0.04%) d 99.99 (99.95%) f 0.00 (0.00%)

(0.96991) LP (3) Mn1
s (0.38%) p 1.24 (0.47%) d 99.99 (99.16%) f 0.00 (0.00%)

(0.96726) LP (4) Mn1
s (0.43%) p 0.80 (0.34%) d 99.99 (99.22%) f 0.00 (0.00%)

(0.96511) LP (5) Mn1
s (0.04%) p 3.97 (0.17%) d 99.99 (99.79%) f 0.00 (0.00%)

Lone pairs of Mn atoms and bonding orbitals (Beta Molecular Orbital, BMO) between selected atoms from NBO analysis in **3**:

(0.91611) BD (1) Mn1-Mn121
(50.26%) 0.7089*Mn1 s (60.55%) p 0.30 (18.13%) d 0.35 (21.32%) f 0.00 (0.00%)
(49.74%) 0.7053*Mn121 s (81.80%) p 0.19 (15.83%) d 0.03 (2.37%) f 0.00 (0.00%)

(0.97645) LP (1) Mn121
s (0.12%) p 4.23 (0.49%) d 99.99 (99.40%) f 0.00 (0.00%)

(0.97317) LP (2) Mn121
s (0.01%) p 1.00 (0.04%) d 99.99 (99.96%) f 0.00 (0.00%)

(0.96977) LP (3) Mn121
s (0.37%) p 1.30 (0.48%) d 99.99 (99.16%) f 0.00 (0.00%)

(0.96698) LP (4) Mn121
s (0.46%) p 0.76 (0.35%) d 99.99 (99.19%) f 0.00 (0.00%)

(0.96502) LP (5) Mn121
s (0.04%) p 4.17 (0.17%) d 99.99 (99.79%) f 0.00 (0.00%)

Table S6: Computed MOs for **3**.

AMO	BMO
HOMO-25	
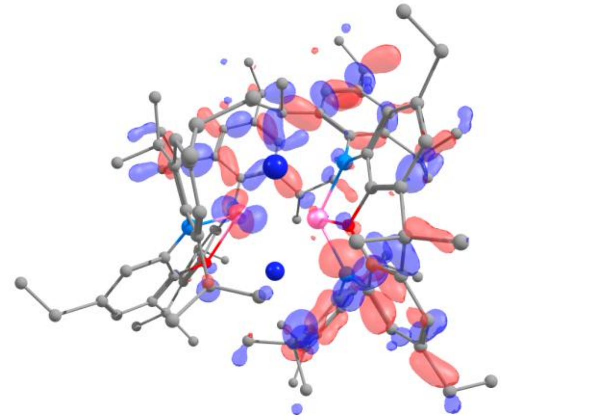	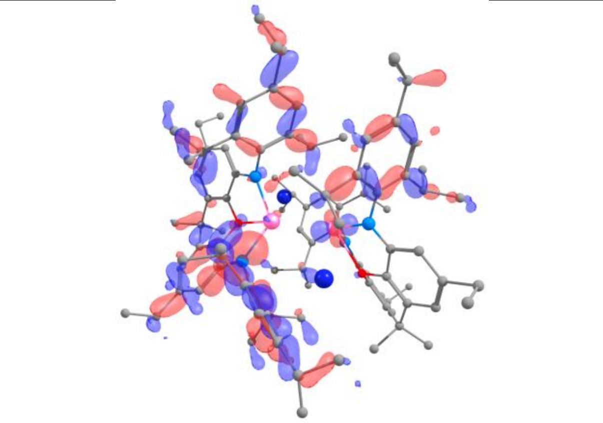
HOMO-24	
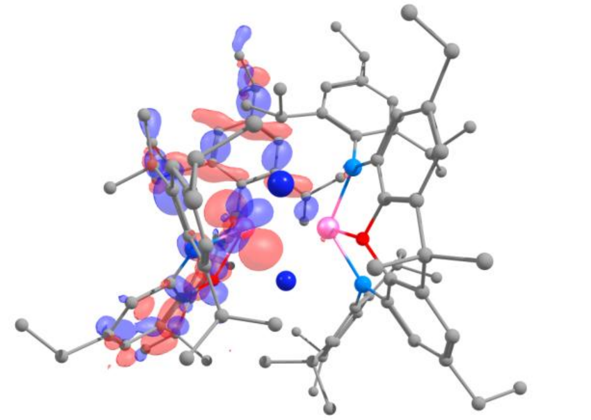	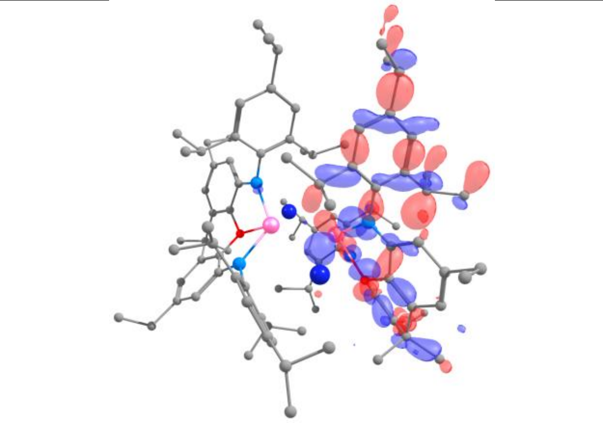
HOMO-22	
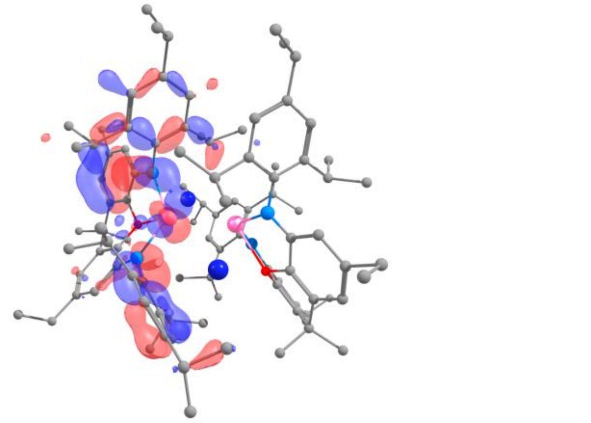	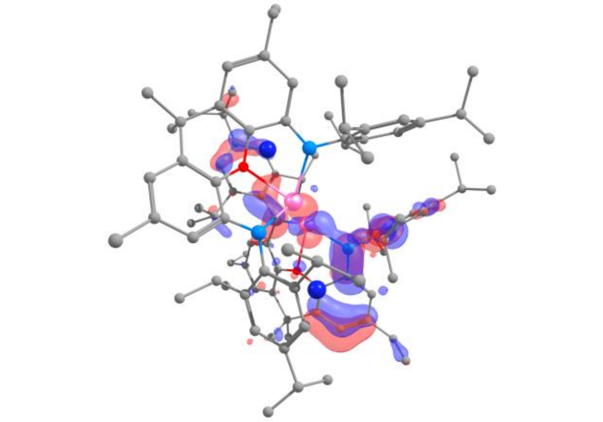

Table S6 (continued): Computed MOs for **3**.

AMO	BMO
HOMO-18	
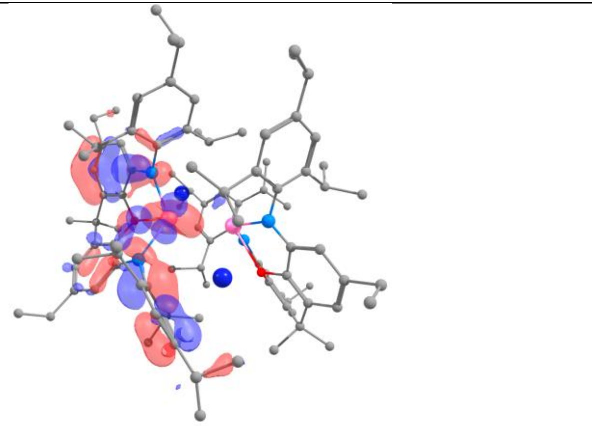	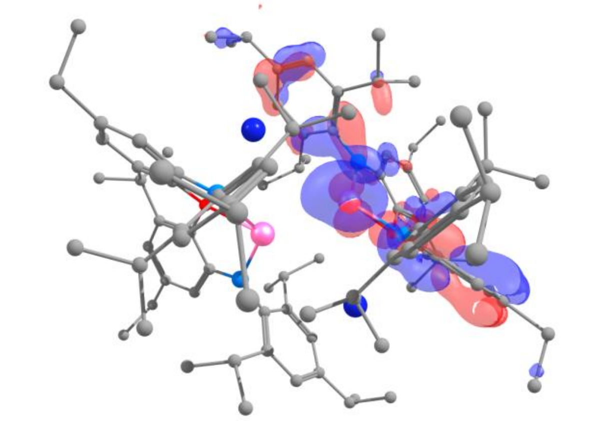
HOMO-17	
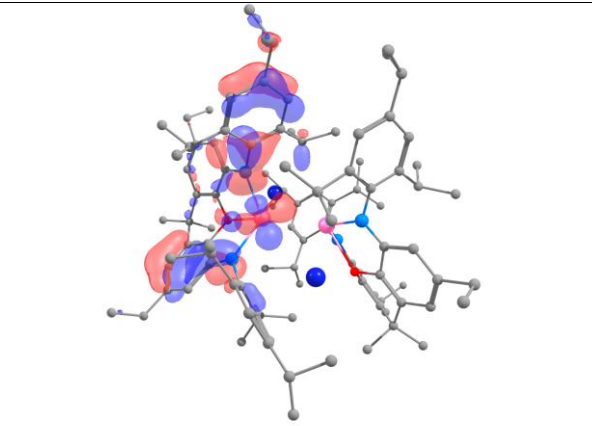	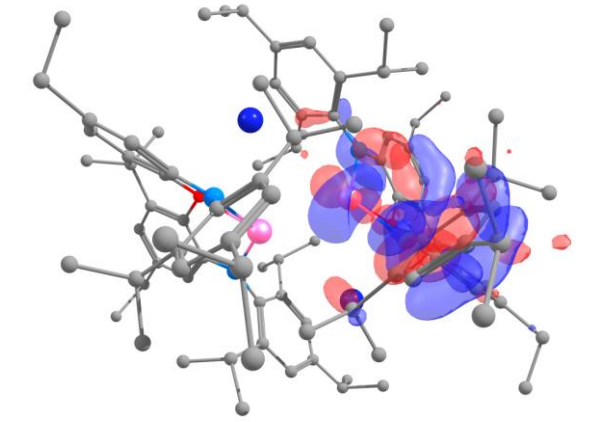

The orbital energy difference between two alpha and beta d-orbitals (HOMO-25 and HOMO-24) are negligible whereas the other three d-orbitals (HOMO-22, HOMO-18 and HOMO-17) are 200 cm^{-1} apart implying the presence of 6 unpaired electrons.

Table S7: Energetics for different spin states computed for [(K(^{TCHP}NON)Mn)$_2$(μ-N_2)], **4**.

Spin states	ΔE (kcal/mol)
S=4	0.0
S=5	23.9
S=6	34.8

Table S8: DFT computed spin densities on selected atoms in **4** (S=4).

Atom labels	Spin densities
Mn1	4.529623
Mn2	4.486929
N3	-0.638577
N4	-0.633267
K5	0.034246
K6	0.041951

Cartesian coordinates (Å) for the optimised theoretical structures can be found in a separate xyz file.

Literature

[1] D. F. Evans, *J. Chem. Soc.* **1959**, 2003-2005.

[2] G. A. Bain, J. F. Berry, *J. Chem. Educ.* **2008**, *85*, 532.

[3] R. Mondal, K. Yuvaraj, T. Rajeshkumar, L. Maron, C. Jones, *Chem. Commun.* **2022**, *58*, 12665-12668.

[4] R. Mondal, M. J. Evans, D. T. Nguyen, T. Rajeshkumar, L. Maron, C. Jones, *Chem. Commun.* **2024**, *60*, 1016-1019.

[5] J. Hicks, M. Juckel, A. Paparo, D. Dange, C. Jones, *Organometallics* **2018**, *37*, 4810-4813.

[6] R. E. H. Kuveke, L. Barwise, Y. van Ingen, K. Vashisth, N. Roberts, S. S. Chitnis, J. L. Dutton, C. D. Martin, R. L. Melen, *ACS Cent. Sci.* **2022**, *8*, 855-863.

[7] N. F. Chilton, R. P. Anderson, L. D. Turner, A. Soncini, K. S. Murray, *J. Comput. Chem.* **2013**, *34*, 1164-1175.

[8] T. M. McPhillips, S. E. McPhillips, H.-J. Chiu, A. E. Cohen, A. M. Deacon, P. J. Ellis, E. Garman, A. Gonzalez, N. K. Sauter, R. P. Phizackerley, S. M. Soltis, P. Kuhn, *J. Synchrotron Radiat.* **2002**, *9*, 401-406.

[9] W. Kabsch, *J. Appl. Crystallogr.* **1993**, *26*, 795-800.

[10] G. Sheldrick, *Acta Crystallogr. A* **2015**, *71*, 3-8.

[11] G. Sheldrick, *Acta Crystallogr. C* **2015**, *71*, 3-8.

[12] C. B. Hubschle, G. M. Sheldrick, B. Dittrich, *J. Appl. Crystallogr.* **2011**, *44*, 1281-1284.

[13] A. Spek, *Acta Crystallogr. D* **2009**, *65*, 148-155.

[14] A. D. Becke, *J. Chem. Phys*. **1993**, 98, 5648.

[15] (a) D. Andrae, U. Häussermann, M. Dolg, H. Stoll and H. Preuss, *Theor. Chim. Acta*. **1990**, 77, 123; (b) J. M. L. Martin, A. Sundermann, *J. Chem. Phys.* **2001**, 114, 3408.

[16] (a) R. Ditchfield, W. J. Hehre and J. A. Pople, *J. Chem. Phys.* **1971**, 54, 724; (b) W. J. Hehre, R. Ditchfield and J. A. Pople, *J. Chem. Phys.* **1972**, 56, 2257; (c) P. C. Hariharan and J. A. Pople, *Theor. Chem. Acc.* **1973**, 28, 213; (d) R. Krishnan, J. S. Binkley, R. Seeger, J.A. Pople, *J. Chem. Phys*. **1980**, 72, 650.

[17] S. Grimme, S. Ehrlich, L. Goerigk, *J. Comp. Chem.* **2011**, 32, 1456.

[18] Gaussian 09, Revision D.01: M. J. Frisch, G. W. Trucks, H. B. Schlegel, G. E. Scuseria, M. A. Robb, J. R. Cheesman, G. Scalmani, V. Barone, B. Mennucci, G. A. Petersson, H. Nakatsuji, M. Caricato, X. Li, H. P. Hratchian, A. F. Izmaylov, J. Bloino, G. Zheng, J. L. Sonnenberg, M. Hada, M. Ehara, K. Toyota, R. Fukuda, J. Hasegawa, M. Ishida, T. Nakajima, Y. Honda, O. Kitao, H. Nakai, T. Vreven, J. A. Jr. Montgomery, J. E. Peralta, F. Ogliaro, M. Bearpark, J. J. Heyd, E. Brothers, K. N. Kudin, V. N. Staroverov, R. Kobayashi, J. Normand, K. Raghavachari, J. C. Burant, S. S. Iyengar, J. Tomasi, M. Cossi, N. Rega, M. J. Millam, M. Klene, J. E. Knox, J. B. Cross, V. Bakken, C. Adamo, J. Jaramillo, R. Gomperts, R. E. Stratmann, O. Yazyev, A. J. Austin, R. Cammi, C. Pomelli, J. W. Ochterski, R. L. Martin, K. Morokuma, V. G. Zakrzewski, G. A. Voth, P. Salvador, J. J. Dannenberg, S. Dapprich, A. D. Daniels, O. Farkas, J. B. Foresman, J. V. Ortiz, J. Cioslowski and D. J. Fox, Gaussian Inc., **2009**, Wallingford CT.
